# Self-Adaptive AdamW-Guided Optimization: A Learning-Driven Metaheuristic for Solving Complex Real-World Engineering Problems

**DOI:** 10.3390/e28060660

**Published:** 2026-06-09

**Authors:** Yuhang Xie, Wei Li, Cheng Zhong, Shang Gao, Kai Xu, Juanjuan Tu, Bin Qin

**Affiliations:** 1 School of Computer, Jiangsu University of Science and Technology, Zhenjiang 212003, China; 232211901130@stu.just.edu.cn (Y.X.);; 2 Department of Power Engineering, PLA Army Engineering University, Nanjing 210007, China

**Keywords:** AdamW, benchmark test suite, engineering design optimization, pseudo-gradients, metaheuristic optimization

## Abstract

Given the growing complexity of continuous optimization problems in strongly coupled and black-box environments, this study proposes a novel adaptive gradient-guided metaheuristic, referred to as Self-Adaptive AdamW-Guided Optimization (SAWG). Without requiring explicit gradient information, SAWG constructs population-based pseudo-gradients and systematically integrates key AdamW mechanisms, including adaptive moment estimation, step-size regulation, and weight decay, to guide efficient population updates. Furthermore, a stagnation-aware adaptive control strategy is introduced to alleviate premature convergence and dynamically balance exploration and exploitation. To evaluate the optimization performance of SAWG, experiments were conducted on the CEC2017 and CEC2020 benchmark suites and eight engineering optimization problems. SAWG was also compared with nine other typical and novel high-performance optimizers. Experimental results and statistical analysis show that SAWG achieved excellent optimization performance in most test tasks and maintained strong adaptability and competitiveness in various numerical optimization problems. Therefore, SAWG can be regarded as a high-performance optimizer, providing a novel and effective method for solving complex numerical optimization tasks.

## 1. Introduction

Many critical tasks arising in engineering design, intelligent manufacturing, and energy scheduling [1] can be abstracted as complex continuous or hybrid optimization problems [2,3]. These problems are commonly associated with substantial nonlinearity, strong inter-variable coupling, and black-box characteristics [4], which collectively constrain the applicability of traditional gradient-based optimizers [5]. Since metaheuristic algorithms do not require an explicit analytical form of the objective function or accessible gradient information [6], they have become an important methodological paradigm for tackling such problems [7]. However, the No Free Lunch theorem [8] implies that no universal optimizer can maintain consistent superiority across all classes of optimization tasks, thereby underscoring the necessity of constructing specialized search mechanisms in accordance with problem-specific structural characteristics.

Although metaheuristic algorithms have progressed considerably, three fundamental difficulties still constrain their performance [9] on high-dimensional constrained optimization problems with complex structures. First, when the search process relies excessively on the current best individual or local refinement operators, population diversity is often diminished rapidly, thereby increasing the risk of premature convergence [10,11]; Ren et al. showed that maintaining diversity provably benefits evolutionary multimodal optimization [12]. Second, the coordination between exploration and exploitation is commonly regulated by fixed empirical parameters rather than mechanisms that respond adaptively to the search state and problem structure, making late-stage stagnation a recurrent issue [13]. Third, when the updating process is driven predominantly by stochastic perturbations, the resulting search trajectory may exhibit pronounced oscillatory behavior in complex spaces characterized by substantial scale heterogeneity, consequently undermining both convergence efficiency and overall stability [14,15].

Inspired by parameter optimization mechanisms in machine learning, Doerr and Zheng theoretically analyzed multi-objective evolutionary algorithms on multimodal objectives and showed that heavy-tailed mutation and stagnation-aware strategies can substantially improve search efficiency [16]; furthermore, Li et al. also demonstrated the effectiveness of restarting and search [17]. The present study proposes a metaheuristic framework, denoted as Self-Adaptive AdamW-Guided Optimization (SAWG), that synthesizes a stagnation-aware control mechanism, AdamW-based updating principles, and a pseudo-gradient-driven search [18] strategy. Specifically, SAWG constructs pseudo-gradient signals to impose globally directional guidance on population evolution, while first-order momentum and second-order statistical estimation are jointly exploited to adaptively modulate the update magnitude and search direction. Furthermore, a decoupled weight decay [15] mechanism is incorporated to reinforce the stability of the search dynamics.

The principal contributions of this study are summarized as follows:

1. This study formulates the Self-Adaptive AdamW-Guided Optimization (SAWG) algorithm under an AdamW-based updating framework. The proposed method systematically integrates momentum memory with second-order statistical estimation and further characterizes the dynamic search behavior of SAWG through visualization-based analyses of population diversity and search trajectories. In doing so, it establishes a methodological framework for population-based search from the perspective of machine-learning-driven optimization.
2. SAWG is systematically evaluated on the CEC 2017 benchmark suite under multiple dimensional settings. The results demonstrate that SAWG achieves stronger overall performance than a range of classical and advanced baseline optimizers across different problem scales, and its statistical superiority is further supported by significance tests. Specifically, SAWG exhibits consistent advantages in Friedman ranking, Wilcoxon tests, and aggregated performance indicators.
3. To examine its practical applicability, SAWG is further deployed on eight classical engineering optimization problems. The corresponding results show that the proposed method delivers favorable solution quality together with stable performance across all considered engineering tasks, thereby substantiating its robustness and engineering relevance in complex real-world environments.

The remainder of this paper is organized as follows. Section 2 discusses the motivation underlying the proposed algorithm. Section 3 introduces the overall framework and core mechanisms of SAWG. Section 4 presents the experimental setup and the associated result analysis. Section 5 reports the outcomes on engineering applications. Section 6 concludes the paper and outlines possible directions for future research.

## 2. Related Work

To articulate the design motivation of the proposed SAWG algorithm, this section contrasts gradient-driven optimization with metaheuristic search from the perspective of optimization paradigms. We first review the core concepts and prerequisites of gradient-based methods, followed by an analysis of the advantages and inherent limitations of metaheuristics in black-box scenarios. Finally, we summarize recent advances in directional search mechanisms under derivative-free [18,19] conditions, highlighting the lack of systematic integration of mature gradient techniques, which lays the methodological foundation for SAWG.

### 2.1. Gradient-Based Optimization in Machine Learning

Gradient-based optimization serves as the computational foundation of machine learning, relying on the assumption that the objective function is differentiable or admits reliable gradient approximations. Under this framework, advanced optimizers introduce momentum to suppress high-frequency oscillations [14], adaptive learning rates to mitigate scale inconsistencies, and weight decay to enhance generalization; for instance, Loshchilov and Hutter proposed AdamW, Kunstner et al. analyzed why Adam can outperform gradient descent in language models, and Zhou et al. further studied the convergence and generalization behavior of AdamW [20,21]. However, the efficacy of these methods strictly depends on gradient availability, rendering them inapplicable to complex scenarios where analytical derivatives are inaccessible or discontinuous.

### 2.2. Metaheuristic Algorithms for Black-Box Optimization

For non-differentiable or computationally expensive black-box problems, metaheuristic algorithms perform global exploration through heuristic rules [22] and stochastic perturbations. Requiring minimal mathematical assumptions, they exhibit strong adaptability in complex landscapes; Hüttenrauch and Neumann developed a robust black-box optimization framework, and Zhang et al. systematically benchmarked zeroth-order optimization as a gradient-free alternative [23]. Nevertheless, they are heavily reliant on randomness and often lack explicit directional constraints, making convergence susceptible to oscillation or stagnation in high-dimensional spaces [24,25]. Furthermore, balancing exploration and exploitation typically relies on empirical parameters, hindering adaptive dynamic adjustments based on search states; related recent work on task-dependent optimizer generation and history-aware black-box optimization also reflects this limitation [26].

### 2.3. Bridging Gradient-Based Learning and Metaheuristic Search

Although gradient-based learning and metaheuristic search are traditionally viewed as distinct branches, recent studies have attempted to incorporate directional or memory-based mechanisms into derivative-free frameworks. Existing efforts include gradient matching for offline black-box optimization [19] by Hoang et al., pseudo-zeroth-order directional construction by Yue et al., and reinforced in-context black-box optimization by Song et al., all of which improve search behavior to some extent [18,27,28]. However, these methods primarily focus on local or partial modifications and fail to systematically integrate mature strategies from gradient optimizers, such as momentum, second-order moment estimation, and decoupled regularization, from a dynamics perspective [20,21]. How to embed these mechanisms into a derivative-free framework to construct a stable and memory-guided search process remains a critical open problem that this study aims to address.

## 3. SAWG Methodology

Based on the above analysis, this section presents the methodological foundation and overall framework of the proposed Self-Adaptive AdamW-Guided Optimization (SAWG). The core objective of SAWG is to construct a swarm-based search mechanism endowed with gradient-learning characteristics under gradient-free black-box optimization settings. To this end, SAWG integrates AdamW-style adaptive update mechanisms, dynamic parameter modulation strategies, and stagnation-aware restart schemes to achieve coordinated global–local regulation of the search process. As a result, the proposed framework enables more stable and efficient optimization behavior when addressing complex, high-dimensional, and nonconvex problems.

### 3.1. Gradient Descent as the Algorithmic Core

Gradient descent (GD) is one of the most fundamental optimization paradigms in continuous optimization and machine learning. Its central idea is to iteratively update parameters along the negative gradient direction of the objective function in the parameter space, thereby gradually approaching the optimal solution. The gradient characterizes the direction of steepest ascent at a given point, and moving in the opposite direction yields the fastest decrease in the objective value. For differentiable objective functions, this mechanism provides a clear, stable, and readily interpretable descent direction.

In modern optimization theory, gradient descent has evolved far beyond its basic form with a fixed learning rate. Tian et al. show that rethinking weight decay in foundation-model fine-tuning can improve robustness and training stability by clarifying how AdamW-style decay affects optimization dynamics [29]. By incorporating mechanisms such as momentum accumulation, adaptive scale adjustment, and weight decay, gradient-based methods have achieved significantly improved robustness and convergence performance in nonconvex, noisy, and high-dimensional optimization scenarios. Optimizers exemplified by AdamW jointly model first-order and second-order statistical information to adaptively regulate both search directions and step sizes, and these approaches are widely regarded as a major milestone in the development of gradient-based optimization methods [20,21].

Consider a continuous optimization problem with a differentiable objective function:

(1) $$\begin{array}{c} \min_{x\in \Omega }f\left(x\right),\;\;\Omega =\left\{x|l\leq x\leq u\right\} \end{array}$$

where $f:R^{d}\to R$ denotes the objective function; $x={\left[x_{1},\ldots ,x_{d}\right]}^{⊤}$ is the decision vector; Ω is a box-constrained feasible region; and $l,u\in R^{d}$ represent the lower and upper bounds, respectively.

If *f* is differentiable over Ω and its gradient is accessible, gradient descent (GD) constructs descent updates via a local first-order approximation. Specifically, for a given iterate $x_{t}$, the first-order Taylor expansion is given by

(2) $$\begin{array}{c} f\left(x_{t}+\Delta x\right)\approx f\left(x_{t}\right)+\nabla f{\left(x_{t}\right)}^{⊤}\Delta x. \end{array}$$

where *t* denotes the iteration index, $x_{t}$ is the current solution, and Δx is the update increment. To achieve the maximum decrease in this approximation under the step-size constraint ${\left|\Delta x\right|}_{2}\leq \eta_{t}$, the following constrained subproblem is formulated:

(3) $$\begin{array}{c} \min_{\Delta x}\;\nabla f{\left(x_{t}\right)}^{⊤}\Delta x\;s.t.\;{∥\Delta x∥}_{2}\leq \eta_{t}. \end{array}$$

By the Cauchy–Schwarz inequality,    

(4) $$\begin{array}{c} \nabla f{\left(x_{t}\right)}^{⊤}\Delta x\geq -∥\nabla f\left(x_{t}\right){∥}_{2}\cdot {∥\Delta x∥}_{2}, \end{array}$$

with equality attained when $\Delta x=-\eta_{t}\frac{\nabla f(x_{t})}{|\nabla f\left(x_{t}\right){|}_{2}}$. Consequently, the direction of steepest descent corresponds to $-\nabla f(x_{t})$, yielding the classical GD update

(5) $$\begin{array}{c} x_{t+1}=x_{t}-\eta_{t}\nabla f\left(x_{t}\right), \end{array}$$

where $\eta_{t}>0$ denotes the learning rate that controls the update magnitude along the descent direction.

Despite its simplicity, GD is often challenged by nonconvex landscapes, noisy gradients, and heterogeneous scaling across dimensions. To address these limitations, modern optimization methods introduce momentum accumulation and second-order moment estimation, leading to adaptive gradient optimizers such as Adam and AdamW. These methods estimate the first-order momentum and second-order statistics of the gradient sequence $g_{t}$, enabling more robust update directions and coordinatewise adaptive step-size adjustment. This principle underpins the design of SAWG: even in black-box optimization settings where true gradients are unavailable, AdamW-level update mechanisms can be effectively exploited by constructing suitable surrogate gradient sequences.

### 3.2. Initialization Strategy

Each search agent is denoted by $x_{ij}$, which represents the position component of the *i*th individual in the *j*th dimension of the search space. All position components are required to satisfy the box constraints

(6) $$\begin{array}{c} l\leq x_{ij}\leq u\;\forall i=1,2,\ldots ,N,\;\forall j=1,2,\ldots ,\mathrm{Dim} \end{array}$$

where *N* denotes the population size and Dim is the dimensionality of the problem. The vectors l and u correspond to the lower and upper bounds of the decision variables, respectively. Consistent with conventional metaheuristic frameworks, SAWG initializes a population of candidate solutions randomly within the prescribed bounds to explore the search space, as given by

(7) $$\begin{array}{c} x_{ij}=\mathrm{rand}\left(N,\mathrm{Dim}\right)\times \left(l-u\right)+l \end{array}$$

where rand(N,Dim) denotes an N×Dim matrix with entries uniformly sampled from the interval [0,1].

At the initialization stage, SAWG identifies the individual with the best fitness value as the elite solution. For minimization problems, the position of this individual provides an approximation of the current global optimum. In parallel, the AdamW momentum states for each individual are initialized as

(8) $$m_{i}^{\left(0\right)}=0_{D},\;v_{i}^{\left(0\right)}=0_{D}.$$

where $0_{D}$ denotes a *D*-dimensional zero vector. This initialization strategy prevents any historical bias at the early search stage, ensuring that the momentum information is accumulated adaptively and driven exclusively by the subsequent optimization process.

### 3.3. Gradient-Enhanced Update Mechanism of SAWG

#### 3.3.1. Pseudo-Gradient Construction

In engineering design optimization and CEC benchmark testing, objective functions are typically treated as black boxes, for which analytical expressions are unavailable or non-differentiable, making the true gradient ∇f(x) inaccessible. To introduce gradient-like directional guidance in such gradient-free settings, SAWG constructs a computable pseudo-gradient by leveraging population-level statistics and information on elite solutions. This design yields a learnable search direction that can be naturally integrated into the subsequent AdamW update framework.

At iteration *t*, the population is denoted by ${\left\{x_{i,t}\right\}}_{i=1}^{N}$, where *N* is the population size, $x_{i,t}\in R^{D}$ represents the position of the *i*th individual, and *D* is the dimensionality of the problem. The population mean is defined as

(9) $${\bar{x}}_{t}=\frac{1}{N}\sum_{j=1}^{N}x_{j,t}.$$

The elite index $j_{t}^{⋆}$ and elite position $x_{t}^{⋆}$ are given by

(10) $$\begin{array}{c} j_{t}^{⋆}=\arg\min_{j\in \{1,\ldots ,N\}}f\left(x_{j,t}\right),\;x_{t}^{⋆}=x_{j_{t}^{⋆},t}. \end{array}$$

For each individual, two fundamental guidance vectors are constructed to provide continuously adjustable directional information between exploration and exploitation. The population-mean-guided vector $P_{i,t}^{\left(1,k\right)}$ emphasizes exploratory and aggregative behavior, whereas the elite-guided vector $P_{i,t}^{\left(2\right)}$ promotes exploitation toward the best-so-far solution:

(11) $$P_{i,t}^{\left(1,k\right)}={\bar{x}}_{t}-{\tilde{x}}_{i,t}^{\left(k\right)},\;P_{i,t}^{\left(2\right)}=x_{t}^{⋆}-x_{i,t}.$$

It should be noted that ${\bar{x}}_{t}$ here comes from Equation (9), representing the overall mean of the *t*th iteration, and $x_{i,t}$ comes from Equation (11), representing the position of the *i*th individual in the *t*th iteration. ${\tilde{x}}_{i,t}^{\left(k\right)}$ represents the *k*th intermediate line state, defined as follows:

(12) $$\begin{array}{c} {\tilde{x}}_{i,t}^{\left(k\right)}=\left\{\begin{array}{cc} \left(\omega_{t}+\alpha_{i}\right)x_{i,t}, & k=1,\\ x_{i,t}+\sin\left(2\pi tD\right){\tilde{x}}_{i,t}^{\left(k-1\right)}, & k>1. \end{array}\right. \end{array}$$

${\tilde{x}}_{i,t}^{\left(k\right)}$ denotes the *k*th intermediate line state constructed within the same generation (k=1,…,K, with K=⌊D/2⌋). The variable $\omega_{t}$ is a time-varying random amplitude scalar, and $\alpha_{i}$ is an individual-dependent phase coefficient, defined as follows:

(13) $$\begin{array}{cc} \; & \omega =u_{t}\left(\frac{t^{2}}{T^{2}}-\frac{2t}{T}+\frac{1}{2}\right),\;\;u_{t}∼U\left(0,1\right), \end{array}$$

(14) $$\begin{array}{cc} \; & \alpha_{i}=\cos\left(2\pi (1-u_{i})\right),\;\;u_{i}∼U\left(0,1\right). \end{array}$$

where T denotes the total number of iterations. To smoothly transition from broad exploration to focused exploitation, SAWG employs a hybrid weighting strategy that couples iteration progress with individual ranking. Let

(15) $$\begin{array}{c} \rho_{i}=\frac{i-1}{N-1}\in \left[0,1\right],\;c_{2}\left(i,t\right)=\frac{t}{T}\rho_{i},\;c_{1}\left(i,t\right)=1-c_{2}\left(i,t\right) \end{array}$$

Then the final guidance vector is obtained as

(16) $${P^{\left(k\right)}}_{i,t}=c_{1}\left(i,t\right)P_{i,t}^{\left(1,k\right)}+c_{2}\left(i,t\right)P_{i,t}^{\left(2\right)}.$$

Before being integrated into AdamW, the guidance vector is normalized to eliminate scale discrepancies and improve numerical stability:

(17) $$P_{i,t}^{\left(\tilde{k}\right)}=\frac{P_{i,t}^{\left(k\right)}}{∥P_{i,t}^{\left(k\right)}∥+\varepsilon }.$$

where ε>0 is a small constant and ||·|| is the Euclidean norm. Finally, the black-box pseudo-gradient is constructed as

(18) $$\begin{array}{c} g_{i,t}=x_{i,t}-C_{t}⊙P_{i,t}^{\left(\tilde{k}\right)},\;C_{t}=1-r,\;r∼U\left(0,1\right) \end{array}$$

where ⊙ denotes elementwise multiplication and $C_{t}$ is a stochastic interaction-strength scalar shared across all dimensions.

Since $c_{2}\left(i,t\right)=\left(t/T\right)\rho_{i}$, the contribution of the elite-guided component is weak in the early iterations and gradually increases as the search proceeds. Therefore, the guidance vector is initially dominated by the population-mean-guided component $P_{i,t}^{\left(1\right)}$, which encourages population-level exploration and reduces the risk of premature convergence. In later iterations, the influence of the elite-guided component $P_{i,t}^{\left(2\right)}$ becomes stronger, especially for lower-ranked individuals, thereby promoting exploitation around promising regions. The variable $\rho_{i}$ prevents all individuals from being attracted to the elite solution with the same intensity, which helps maintain diversity while improving convergence. Combined with the normalization process in Equation (17), the constructed guidance vector provides a smooth transition from global exploration to local exploitation.

It should be emphasized that the pseudo-gradient in SAWG is not a true derivative. Instead, it is generated from the relationship between the current position and the constructed guidance direction, which jointly captures the global population trend and the elite-driven attraction. This design provides continuously adjustable directional information between global exploration and local exploitation. By incorporating normalization and stochastic scaling, the pseudo-gradient maintains directional consistency while avoiding excessive determinism, thereby mitigating premature convergence and offering a unified interface for subsequent momentum integration and adaptive updates.

#### 3.3.2. AdamW-Guided Gradient-Enhanced Update

After the pseudo-gradient $g_{i,t}$ is obtained, SAWG adopts the AdamW momentum integration mechanism to smooth the driving signal and introduce scale adaptivity, thereby constructing a gradient-type update structure under black-box optimization settings. For each individual, first-order and second-order momentum terms are maintained and updated according to

(19) $$\begin{array}{c} m_{i,t}=\beta_{1}m_{i,t-1}+\left(1-\beta_{1}\right)g_{i,t}, \end{array}$$

(20) $$\begin{array}{c} v_{i,t}=\beta_{2}v_{i,t-1}+\left(1-\beta_{2}\right)g_{i,t}^{⊙2} \end{array}$$

where $\beta_{1},\beta_{2}\in \left(0,1\right)$ are the momentum decay coefficients (set to $\beta_{1}=0.9$ and $\beta_{2}=0.999$), while $g_{i,t}$ denotes the pseudo-gradient defined in Equation (20).

To correct the bias introduced during the early iterations, bias-corrected estimates are computed as

(21) $$\begin{array}{c} {\hat{m}}_{i,t}=\frac{m_{i,t}}{1-\beta_{1}^{t}},\;{\hat{v}}_{i,t}=\frac{v_{i,t}}{1-\beta_{2}^{t}}. \end{array}$$

The resulting adaptive update takes the form

(22) $$\begin{array}{c} x_{t+1}=x_{t}-\eta_{t}\frac{{\hat{m}}_{t}}{\sqrt{{\hat{v}}_{t}}+\varepsilon }, \end{array}$$

where ε is a small constant for numerical stability and $\eta_{t}$ is the learning rate. Building upon this formulation, AdamW introduces decoupled weight decay [20] to prevent the regularization term from being suppressed by second-order adaptive scaling. The corresponding AdamW-style update step is given by

(23) $$\begin{array}{c} \Delta x_{i,t}=\eta_{t}\left(\frac{{\hat{m}}_{i,t}}{\sqrt{{\hat{v}}_{i,t}}+\varepsilon }+\lambda_{t}x_{i,t}\right). \end{array}$$

To achieve adaptive regulation of the search scale, SAWG applies a cosine annealing strategy to both the learning rate $\eta_{t}$ and the weight decay coefficient $\lambda_{t}$. Specifically, $\lambda_{t0}$ is initialized to 0.01, and a piecewise strategy with $\eta_{t}=\eta_{\max}$ is adopted when t<0.4T:

(24) $$\begin{array}{cc} \eta_{t} & =\eta_{\min}+\frac{1}{2}\left(\eta_{\max}-\eta_{\min}\right)\left(1+\cos\frac{\pi t}{T}\right) \end{array}$$

(25) $$\begin{array}{cc} \lambda_{t} & =\lambda_{t0}\cdot 0.5\cdot (1+\cos\left(\frac{\pi \cdot t}{T}\right)). \end{array}$$

where $\eta_{\max}$ and $\eta_{\min}$ denote the upper and lower bounds of the learning rate, respectively, and *T* is the maximum number of iterations. This design enables the algorithm to maintain large update steps in the early stages to enhance exploration, while gradually reducing the step size in later stages to improve solution precision. Finally, the guidance point $G_{i,t}$, influenced by both the adaptive learning rate and the best-performing unit, is defined as

(26) $$G_{i,t}^{\left(k\right)}=x_{i,t}^{\left(k\right)}-\Delta x_{i,t}^{\left(k\right)}.$$

#### 3.3.3. Dynamic Gradient Interaction System

After the guidance point $G_{i,t}$ is obtained, SAWG further incorporates differential interactions to enhance information exchange within the population. Two indices $a_{1},a_{2}\in 1,\ldots ,N\setminus i$ are randomly selected with $a_{1}\neq a_{2}$, and a sign-based interaction intensity is defined as

(27) $$\begin{array}{c} \zeta_{i,t}=\frac{f\left(x_{a_{1},t}\right)-f\left(x_{i,t}\right)}{|f\left(x_{a_{1},t}\right)-f\left(x_{i,t}\right)|+\varepsilon }. \end{array}$$

This coefficient can be regarded as a smoothed sign function of the fitness difference. For minimization problems, when $f\left(x_{a_{1},t}\right)>f\left(x_{i,t}\right)$, the reference individual $x_{a_{1},t}$ is worse than the current individual and $\zeta_{i,t}\approx +1$. Conversely, when $f\left(x_{a_{1},t}\right)<f\left(x_{i,t}\right)$, the reference individual is better and $\zeta_{i,t}\approx -1$. When the two fitness values are nearly identical, the small constant ε avoids division by zero and smooths the sign response. Therefore, $\zeta_{i,t}$ is used only to control the orientation of the interaction term, rather than to encode the magnitude of the fitness difference.

On the basis of the guidance point $G_{i,t}$, two complementary candidate generation schemes are constructed:

(28) $$\left\{\begin{array}{c} y_{i,t}^{\left(a,k\right)}={\tilde{x}}_{i,t}^{\left(k\right)}+\zeta_{i,t}\;a_{t}⊙\left(G_{i,t}^{\left(k\right)}-x_{a_{1},t}\right)-a_{t}⊙\left({\tilde{x}}_{i,t}^{\left(k\right)}-x_{a_{2},t}\right),\\ y_{i,t}^{\left(b,k\right)}=x_{a_{1},t}+a_{t}⊙\left(G_{i,t}^{\left(k\right)}-x_{a_{2},t}\right). \end{array}\right.$$

where ${\tilde{x}}_{i,t}^{\left(k\right)}$ comes from Equation (12) and $a_{t}=\left(1-\frac{t}{T}\right)\cdot \mathrm{rand}\left(1,\dim\right)$ is a random perturbation vector modulated by the dynamic scaling factor $\left(1-\frac{t}{T}\right)$, yielding a range of $\left[0,1-\frac{t}{T}\right]$. The candidate $y_{i,t}^{\left(a,k\right)}$ corresponds to a double-differential perturbation around the intermediate line state, whereas $y_{i,t}^{\left(b,k\right)}$ represents an AdamW-guided differential update anchored at $x_{a_{1},t}$. These two schemes jointly provide complementary search patterns.

To dynamically combine the two candidates at the dimensional level, the following switching rule is applied for each dimension j∈1,…,D:

(29) $$x_{i,t}^{\mathrm{new}}\left(j\right)=\left\{\begin{array}{cc} y_{i,t}^{\left(b,k\right)}\left(j\right), & \frac{u_{1}\left(j\right)}{k}>u_{2}\left(j\right),\\ y_{i,t}^{\left(a,k\right)}\left(j\right), & \mathrm{otherwise}, \end{array}\right.\;\;u_{1}\left(j\right),u_{2}\left(j\right)∼U\left(0,1\right).$$

where *k* is the same as in Equations (16)–(18), (26) and (28), all derived from Equation (12). As *k* increases, the probability of selecting $y^{\left(b\right)}$ gradually decreases, resulting in a progressive transition from aggressive to more conservative updates. Finally, boundary handling and greedy acceptance are employed. The newly generated solution is projected onto the feasible domain, and greedy replacement is performed if improvement is achieved:

(30) $$\begin{array}{c} x_{i,t}^{\mathrm{new}}\leftarrow \min\left(\max\left(x_{i,t}^{\mathrm{new}},l\right),u\right). \end{array}$$

#### 3.3.4. Stagnation Detection and Adaptive Restart Mechanism

To mitigate premature convergence induced by prolonged stagnation, SAWG monitors the search dynamics through the relative improvement rate of the global best solution:

(31) $$\begin{array}{c} {\mathrm{RI}}_{t}=\frac{f_{t-1}^{⋆}-f_{t}^{⋆}}{|f_{t-1}^{⋆}|+\varepsilon }, \end{array}$$

where $f_{t}^{⋆}=\min_{i}f\left(x_{i,t}\right)$ denotes the best fitness value at iteration *t*. When ${\mathrm{RI}}_{t}$ remains below a predefined stagnation threshold τ for a consecutive number of iterations, a restart mechanism is activated. Specifically, a proportion γ of the worst-ranked individuals (set to 0.2N in practice) are selected to undergo an opposition-based restart:

(32) $$\begin{array}{c} x_{i,t}\leftarrow l+u-x_{i,t}+\delta ⊙\left(u-l\right), \end{array}$$

where δ is a perturbation vector (to be consistent with the implementation, $\delta ∼N(0,0.1^{2}I)$ can be used). Meanwhile, the corresponding momentum states are reset as

(33) $$\begin{array}{c} m_{i,t}=0,\;v_{i,t}=0. \end{array}$$

By eliminating the influence of historical momentum, this mechanism prevents negative transfer to newly explored regions, thereby restoring population diversity and improving the robustness of the search process.

#### 3.3.5. Lévy Flight–Based Global Exploration Compensation

Although the dynamic interaction mechanism improves local exploitation and information exchange, premature convergence may still arise in complex multimodal black-box optimization problems. To compensate for insufficient global exploration, SAWG probabilistically activates long-range Lévy jumps at the end of each generation with probability $p_{t}$:

(34) $$\begin{array}{c} p_{t}=p_{\min}+\left(p_{\max}-p_{\min}\right)p_{0}\left(t\right), \end{array}$$

(35) $$\begin{array}{c} p_{0}\left(t\right)=\frac{1}{1+\exp\left(-s(\frac{t}{T}-\mu )\right)}. \end{array}$$

where $p_{\min}$ and $p_{\max}$ denote the lower and upper bounds of the triggering probability, while *s* and μ control the inflection point and steepness of the transition curve. Upon activation, a Lévy step vector $L_{i,t}∼\mathrm{Levy}\left(\beta \right)$ is generated, and a candidate solution is constructed as

(36) $$\begin{array}{c} x_{i,t}^{\prime }=x_{t}^{⋆}+L_{i,t}⊙\xi_{t}⊙\left(x_{t}^{⋆}-\frac{2t}{T}x_{i,t}\right), \end{array}$$

where $\xi_{t}$ is a time-varying random coefficient and β is the Lévy distribution index. The Lévy random vector can be generated using the Mantegna method:

(37) $$\begin{array}{c} \sigma_{u}={\left[\frac{\Gamma (1+\beta )\sin(\pi \beta /2)}{\Gamma \left(\frac{1+\beta }{2}\right)\beta 2^{(\beta -1)/2}}\right]}^{1/\beta }, \end{array}$$

(38) $$\begin{array}{c} u∼N{\left(0,\sigma_{u}^{2}\right)}_{n\times m},\;v∼N{\left(0,1\right)}_{n\times m},\;\mathrm{Levy}\left(N,D,\beta \right)=\frac{u}{{\left|v\right|}^{1/\beta }}. \end{array}$$

Boundary clipping and greedy acceptance are subsequently applied to $x_{i,t}^{\prime }$, which injects long-tailed jump behavior while preserving the overall convergence tendency. This mechanism significantly increases the probability of escaping local optima and strengthens the global exploration capability. The pseudo-code of the proposed SAWG algorithm is displayed in Algorithm 1.

### 3.4. Time Complexity

Let *N* denote the population size, *D* the problem dimensionality, and *T* the maximum number of outer iterations. The time complexity of SAWG is analyzed primarily from the perspective of its internal algorithmic operators.

- Initialization: Initializing *N* individuals in a *D*-dimensional search space requires a computational cost of O(ND). This step is executed only once, at the beginning of the algorithm.
- Recursive intermediate line-state update: In each outer iteration, every individual performs a recursive intermediate line-state update. The number of inner recursive refinements is $K=\left\lfloor \frac{D}{2}\right\rfloor$. Since each recursive refinement involves *D*-dimensional vector operations, the computational complexity of the recursive update for a single individual is $O\left(KD\right)=O\left(D^{2}\right)$. Accordingly, for the entire population, the complexity of this component in each outer iteration is $O(ND^{2})$.
- Pseudo-gradient and AdamW-guided update: The pseudo-gradient construction, guidance-vector computation, AdamW-style first- and second-order moment updates, weight decay, and candidate-solution generation all involve *D*-dimensional vector operations and are embedded within the recursive intermediate line-state update process. Therefore, the computational cost of this component is already included in $O(ND^{2})$.
- Dynamic interaction and greedy selection: Dynamic differential interaction, boundary handling, and greedy selection are also executed within the inner recursive loop. Since each operation is performed at the level of *D*-dimensional vector computations, their computational costs are likewise included in the main update complexity $O(ND^{2})$.
- Lévy flight compensation: When the Lévy flight compensation mechanism is activated, perturbation updates are applied to *N* individuals in a *D*-dimensional space. Therefore, its worst-case computational complexity is O(ND). Since this term is lower than the main update complexity $O(ND^{2})$, it does not affect the overall dominant complexity.
- Stagnation-aware restart: The stagnation-aware restart mechanism is applied only to a subset of inferior individuals. Let the restart ratio be a constant ρ. The complexity of this component is O(ρND). Since ρ is a constant, this term can be simplified as O(ND), which does not alter the dominant complexity of the algorithm.
- Sorting: At the end of each outer iteration, the population is sorted according to fitness values. The corresponding computational complexity is O(NlogN).

**Algorithm 1** Pseudo-code of SAWG.
**Require:** Population size *N*, maximum number of iterations *T*, lower bound lb, upper       bound ub, problem dimension dim, objective function $f_{obj}$**Ensure:** Best solution position $p_{best}$, best fitness value $f_{best}$, convergence curve cg
  1:Initialize population *X* and first- and second-order moment vectors *m* and *v*  2:Evaluate fitness of each individual and identify the global best solution $p_{best}$  3:Initialize stagnation counter and convergence curve cg  4:**while** t≤T **do**  5:    Update learning rate and weight decay using cosine annealing using Equations (24)     and (25)  6:    Generate dynamic control parameters using Equations (13) and (14)  7:    **for** i=1 to *N* **do**  8:        **for** k=1 to dim/2 **do**  9:            Construct perturbed base position using Equation (12)10:            Randomly select two distinct reference individuals using Equation (28)11:            Compute sign-based fitness comparison coefficient using Equation (27)12:            Generate population-guided and elite-guided directions using Equations (11)             and (16)13:            Update position using AdamW-guided pseudo-gradient using Equation (23)14:            Generate candidate solutions and select via greedy rule using Equation (30)15:        **end for**16:    **end for**17:    Perform Lévy-flight-based long jump with a given probability using Equation (36)18:    Update stagnation counter based on relative improvement19:    **if** stagnation criterion is satisfied **then**20:        Restart worst individuals using opposition-based learning using Equations (31)         and (32)21:    **end if**22:    Record the best fitness value in cg(t)23:    t←t+124:
**end while**
25:**return** $p_{best}$, $f_{best}$, cg


Therefore, the time complexity of SAWG in each outer iteration can be expressed as $O(ND^{2}+ND+N\log N)$. For high-dimensional optimization problems, the dominant term is $O(ND^{2})$. After *T* outer iterations, the overall time complexity of SAWG becomes $O(TND^{2})$. This result indicates that the current version of SAWG exhibits quadratic operator-level complexity with respect to the problem dimensionality *D*. This quadratic complexity primarily originates from the recursive intermediate line-state update mechanism, namely, the inner loop $k=1,2,\ldots ,\left\lfloor \frac{D}{2}\right\rfloor$.

Since each inner recursive refinement involves *D*-dimensional vector operations, the update complexity of a single individual reaches $O(D^{2})$, while the per-generation update complexity of the entire population reaches $O(ND^{2})$. This additional computational overhead is introduced by the recursive intermediate line-state update mechanism, which is designed to enhance search-direction construction, improve the stability of pseudo-gradient-guided updates, and achieve a more effective balance between exploration and exploitation in complex search spaces. Consequently, the current version of SAWG is more suitable for optimization scenarios in which search stability, solution quality, and adaptability to complex high-dimensional landscapes are prioritized.

## 4. Numerical Results and Analysis on Benchmark Test Suites

### 4.1. Experimental Study

All numerical experiments were performed in a unified computational environment to ensure reproducibility and fair comparisons. The experimental platform consisted of a 64-bit Windows 11 operating system, an AMD Ryzen 9 7940H processor running at 4.00 GHz with 8 cores and 16 logical threads, and 16 GB of RAM. All algorithms were implemented and visualized using MATLAB R2025b.

To systematically assess the performance of SAWG on classical benchmark optimization problems and to ensure a fair comparison with competing algorithms, experiments were conducted on the CEC2017 [30] and CEC2020 [31] benchmark suites. In accordance with the official CEC evaluation protocol, each test function was independently run 30 times. All algorithms were executed under identical experimental settings, where the maximum number of function evaluations was fixed at 10,000 and the initial population size was set to 30, thereby ensuring the consistency of the evaluation conditions.

The performance of SAWG was evaluated mainly in terms of the mean and standard deviation over 30 independent runs. The mean was used to characterize the overall optimization performance of the algorithm, whereas the standard deviation was adopted to quantify its stability and robustness. For statistical analysis, Wilcoxon signed-rank tests and Friedman ranking analysis were employed to systematically compare the performance differences among the considered algorithms. In terms of result presentation, SAWG was explicitly highlighted in the convergence curves and boxplots, while the best result for each test function was emphasized in the numerical tables, so as to provide a clear and intuitive comparison of the performance discrepancies among different algorithms.

#### 4.1.1. CEC Single-Objective Benchmark Suites

The benchmark suites adopted in this study were released by the IEEE Congress on Evolutionary Computation (CEC). Owing to their complex function landscapes, diverse structural characteristics, and strong ability to characterize the difficulty of high-dimensional optimization problems, these suites have been widely used for performance evaluation of optimization algorithms. In this work, four problem dimensions, namely, 10, 30, 50, and 100, were consistently considered on both benchmark suites to systematically assess the performance of SAWG.

After the exclusion of the unimodal function F2, the CEC2017 suite contains 29 test functions, including two unimodal functions (F1 and F3), seven multimodal functions (F4–F10), 10 hybrid functions (F11–F20), and 10 composition functions (F21–F30). The search range of all decision variables is uniformly defined as [−100, 100].

CEC2020 comprises 10 test functions, including one unimodal function (F1), three multimodal functions (F2–F4), three hybrid functions (F5–F7), and three composition functions (F8–F10). These functions were drawn from representative test instances in CEC2014 and CEC2017, and are therefore commonly regarded as a benchmark suite that is both representative and broadly applicable. Given its relatively compact scale and sufficiently comprehensive coverage of function categories, CEC2020 was further employed in this study to analyze the qualitative search behavior, convergence characteristics, and stability of SAWG, thereby avoiding excessive redundancy in figure presentation. By contrast, the sections on optimization accuracy evaluation and statistical testing fully report the more comprehensive experimental results obtained on CEC2017.

#### 4.1.2. Comparison Algorithms

To comprehensively evaluate the optimization performance of SAWG, it was compared with nine representative optimization algorithms. The parameter settings of all competing methods followed the recommended configurations reported in their original studies to ensure fairness and comparability.

The first category includes well-established swarm intelligence algorithms, namely, particle swarm optimization (PSO) [32], the gray wolf optimizer (GWO) [33], the whale optimization algorithm (WOA) [34], and Harris hawks optimization (HHO) [35]. These methods generally rely on clear bio-inspired search mechanisms and can achieve a reasonable balance between global exploration and local exploitation, making them widely used in continuous optimization tasks.

The second category consists of recently proposed physics- or nature-inspired metaheuristics, including the tornado optimization algorithm (TOC) [36], the snow ablation optimizer (SAO) [37], the rime optimization algorithm (RIME) [38], the phototropism growth algorithm (PGA) [39], and the stellar oscillation optimizer (SOO) [40]. These methods are typically constructed from natural-process-inspired modeling and often exhibit strong adaptive search capability, yielding competitive performance on complex optimization problems.

### 4.2. Qualitative Analysis of SAWG

**Figure 1:**
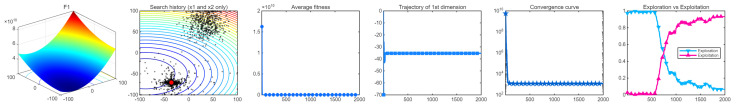
Qualitative analysis of SAWG on unimodal functions with $FE_{\max}=2000$.

**Figure 2:**
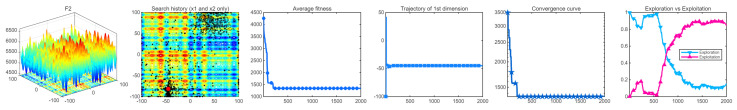
Qualitative analysis of SAWG on multimodal functions with $FE_{\max}=2000$.

**Figure 3:**
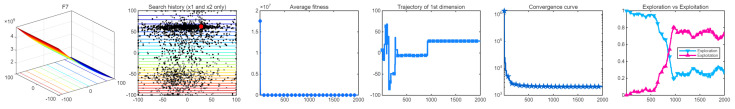
Qualitative analysis of SAWG on hybrid functions with $FE_{\max}=2000$.

**Figure 4:**
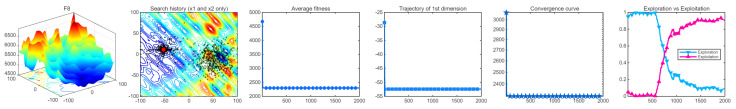
Qualitative analysis of SAWG on composite functions with $FE_{\max}=2000$.

To provide a deeper understanding of the dynamic search behavior of SAWG, a qualitative analysis was conducted on the 10-dimensional CEC2020 benchmark set, as illustrated in Figure 1, Figure 2, Figure 3 and Figure 4. Representative functions from each category were included. Through multiple visualization perspectives, including three-dimensional fitness landscapes, search histories, one-dimensional trajectories, and exploration–exploitation ratios, the search characteristics of the algorithm in various complex spaces were systematically investigated. It should be noted that the purpose of this qualitative study is to reveal the evolutionary dynamics rather than to pursue ultimate numerical precision. Considering that SAWG exhibits rapid convergence in the early stage, the maximum number of function evaluations in this section was reduced to 2000 in order to avoid severe curve overlap in the later stage and to more clearly present its core evolutionary mechanism. It should be noted that this setting was used only for qualitative visualization in this subsection. All quantitative comparisons, including convergence curves, boxplots, numerical tables, and statistical tests, were conducted under the unified budget of 10,000 function evaluations.

The three-dimensional landscapes and search histories indicate that, under the joint effect of pseudo-gradients and adaptive perturbations, SAWG demonstrates strong structured global coverage in the early stage of optimization, thereby reducing the risk of premature stagnation. The average fitness curves and one-dimensional trajectories further show that SAWG maintains a smooth and stable convergence pattern, where performance improvement is driven more by population-level adaptive learning than by the dominance of a few elite individuals. This characteristic enables the algorithm to transition naturally into the later fine exploitation stage and reduces the likelihood of being trapped in local optima. Meanwhile, the exploration–exploitation ratio curves suggest that SAWG can achieve adaptive transitions between search phases under internal feedback, without relying on predesigned fixed scheduling strategies. Overall, this dynamic collaborative mechanism, which spans extensive early exploration, directional convergence in the middle stage, and refined exploitation in the later stage, provides a mechanistic explanation for the favorable stability and robustness of SAWG on complex landscapes, in addition to supporting its strong quantitative performance on CEC2020.

### 4.3. Experimental Results and Discussion on Test Suite

#### 4.3.1. Numerical Results and Accuracy Analysis

The mean values (Mean) and standard deviations (Std) of 10 algorithms under different dimensional settings on the CEC2017 benchmark suite were analyzed. Among the 116 total test cases, SAWG ranked first in 97 cases, including 22 best results under the 10D setting and 25 first-place results each under the 30D, 50D, and 100D settings. These statistics indicate that, as the dimensionality increases, the relative advantage of SAWG is not weakened but instead becomes more pronounced, demonstrating its favorable adaptability to high-dimensional optimization problems. Owing to space limitations, only the results under the 100D setting are presented as a representative example in Table A1.

#### 4.3.2. Convergence Behavior Analysis of SAWG

**Figure 5:**
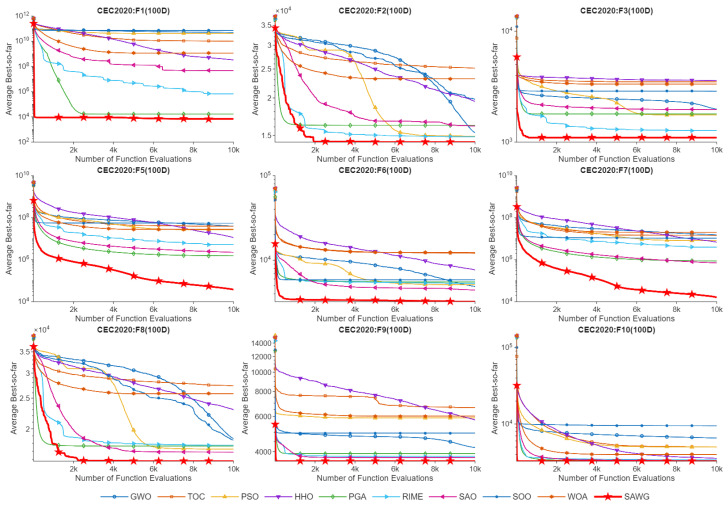
Convergence curves of SAWG and the comparative algorithms on CEC2020 (100D) under $FE_{\max}=10,000$.

This study evaluates the convergence [41] performance of SAWG on unimodal, multimodal, hybrid, and composition functions from the CEC2020 benchmark suite. Consider 100 dimensions as an example. As illustrated in Figure 5, SAWG generally exhibits a faster descending trend, smoother convergence trajectories, and higher solution accuracy across different dimensional settings and landscape complexities, and this advantage becomes more pronounced in high-dimensional scenarios.

On unimodal functions, SAWG is able to approach the global optimum rapidly with fewer function evaluations, reflecting high directional search efficiency. For multimodal functions, its convergence curves maintain an overall continuous downward trend without obvious premature stagnation, indicating a strong ability to escape from local optima. On the more complex hybrid and composition functions, SAWG can effectively overcome the plateau phases in which most competing algorithms tend to become trapped, while continuing to improve solution quality. From the perspective of convergence behavior, this result supports the positive role of its stagnation detection and adaptive restart mechanisms in nonlinear search spaces. Overall, SAWG demonstrates robust convergence behavior, high solution accuracy, and favorable dimensional scalability.

#### 4.3.3. Boxplot Analysis of Results

**Figure 6:**
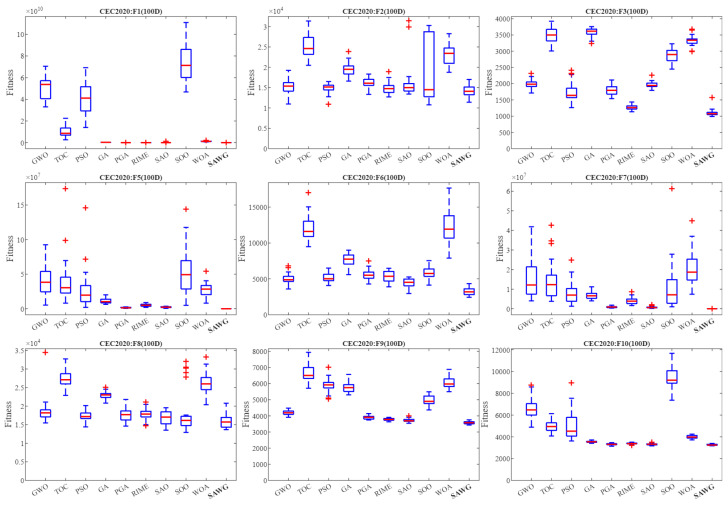
Stability performance of SAWG and the comparative algorithms on CEC2020 (100D) under $FE_{\max}=10,000$.

During the stability analysis stage, boxplots were utilized to assess the stability of the SAWG algorithm, as shown in Figure 6. Each boxplot corresponds to the same experimental conditions and parameter settings as those used in the convergence plots. In the boxplots, the blue rectangle represents the box, the whiskers are displayed using blue dashed lines, and outliers are marked with red “+” symbols. The three horizontal lines inside the box represent, from bottom to top, the first quartile $Q_{1}$, the median $Q_{2}$ (displayed in red), and the third quartile $Q_{3}$. The interquartile range (IQR) is defined as the height of the box, i.e., $|Q_{3}-Q_{1}|$. Outliers are identified as data points below $Q_{1}-1.5\;IQR$ or above $Q_{3}+1.5\;IQR$ [42].

The results show that SAWG generally exhibits lower median values and narrower interquartile ranges (*IQR*s) across different test functions and dimensional settings, directly reflecting its superior solution quality and higher concentration of results. Especially in high-dimensional scenarios, the box associated with SAWG becomes noticeably more compact and contains fewer outliers, indicating that it maintains favorable reliability and search consistency over repeated independent runs. Overall, these statistical distribution characteristics suggest that SAWG is able to achieve a favorable balance between optimization accuracy and search stability in complex search spaces. Its advantage is therefore manifested not only in average optimization metrics but also in the stronger resistance to disturbance reflected by the distribution of its run-level results.

### 4.4. Statistical Tests

**Figure 7:**
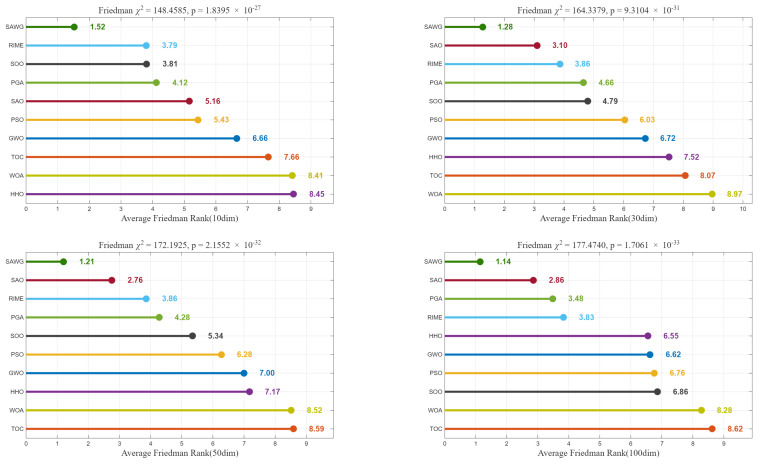
Lollipop charts of SAWG and competing algorithms on the CEC2017 benchmark at 10D, 30D, 50D, and 100D. The top annotation reports the Friedman $\chi^{2}$ statistic and *p*-value. A *p*-value < 0.05 indicates statistically significant differences among the compared algorithms, while the Friedman $\chi^{2}$ quantifies the overall extent of ranking differences.

**Figure 8:**
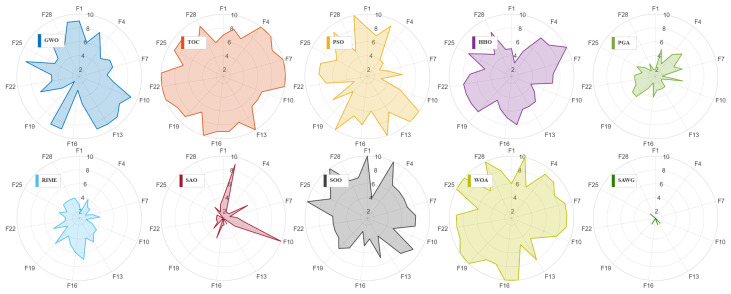
Radar charts of the CEC2017 test suite at 100D. As the benchmark includes 29 test functions, only one label is shown for every three functions on the radar charts to improve readability.

On the CEC2017 benchmark suite, the overall and per-function Friedman ranking results of SAWG and the competing algorithms are visualized as lollipop charts and radar charts, as shown in Figure 7 and Figure 8, respectively. In the radar charts, the radial coordinate represents the Friedman ranking of an algorithm on the corresponding test function, where a position closer to the chart center indicates a better ranking. Meanwhile, the size of the enclosed radar region reflects the overall ranking behavior of the algorithm. A smaller region indicates that the algorithm maintains more stable rankings across different functions and achieves a stronger overall performance.

By contrast, the lollipop charts directly present the overall Friedman ranking results on the entire benchmark suite. In these charts, a shorter bar length corresponds to a better Friedman ranking, thereby providing a concise and intuitive way to compare the global performance of different algorithms.

#### 4.4.1. Friedman Overall Ranking Analysis

The statistical results show that the Friedman tests at all dimensional settings yield extremely small *p*-values (*p* ≪ 0.05), indicating significant performance differences among the compared algorithms. On this basis, SAWG achieves the lowest average Friedman rank across all tested dimensions, showing a clear performance gap over the competing methods. Specifically, in low- and medium-dimensional scenarios, the average rank of SAWG is close to the theoretical optimum of 1. Even in the more challenging high-dimensional cases, its rank changes only slightly and consistently remains in the leading position. As can be seen from the Figure 7, it is worth noting that from 10 dimensions to 100 dimensions, the Friedman score of SAWG decreased from 1.52 to 1.14, indicating that the algorithm becomes more competitive as the dimensional increases.

#### 4.4.2. Performance Analysis of Friedman Individuals

Consider 100 dimensions as an example. Figure 8 illustrate the per-function Friedman rankings of different algorithms on the CEC2017 test suite using radar charts. The results indicate that the radar region associated with SAWG is overall more compact, with most rankings consistently concentrated near the inner circle, suggesting that SAWG possesses strong overall robustness across different problem structures.

On low-complexity functions and several multimodal functions (e.g., Figure 8), SAWG generally maintains the best or second-best ranking, outperforming competing algorithms such as GWO and PSO that exhibit larger fluctuations. This indicates that SAWG can establish effective search directions rapidly while maintaining a favorable balance between convergence efficiency and search stability. On more complex multimodal functions, the rankings of most competing algorithms expand noticeably outward, whereas SAWG still preserves a relatively stable inner-circle distribution, suggesting that it can maintain desirable search stability even in complicated search spaces.

Overall, these statistical results suggest that the performance advantage of SAWG does not rely on specific function types or dimensional settings but is instead reflected in stable and broadly applicable optimization performance across diverse test scenarios.

#### 4.4.3. Wilcoxon Signed-Rank Test

To systematically assess the performance advantage of SAWG, Table 1 and Table 2 summarize the results of the Wilcoxon signed-rank test and Holm multiple-comparison correction on the CEC2017 and CEC2020 benchmark suites, respectively. The significance level was uniformly set to 0.05, where “+”, “≈”, and “−” indicate significant superiority, no significant difference, and significant inferiority, respectively.

The statistical results show that, on unimodal functions where algorithmic performance tends to be relatively similar, SAWG generally maintains performance comparable to or slightly better than that of mainstream algorithms. By contrast, in more challenging optimization scenarios characterized by high dimensionality, nonconvexity, and multimodality, SAWG obtains a larger number of statistically significant advantages (“+”). This suggests that the core search mechanism of SAWG possesses favorable scalability and stability when handling complex nonlinear search spaces. However, it is undeniable that recent algorithms (SOO, PGA, RIME, etc.) are also quite competitive in low-dimensional environments. Overall, these statistical tests not only support the competitive advantage of SAWG in terms of overall optimization performance but also indicate, from a statistical perspective, that its performance gains remain relatively stable and consistent across different problem structures and dimensional settings.

## 5. Application of SAWG to Real-World Problems

This section compares SAWG with nine competing algorithms on eight real-world constrained engineering optimization problems, aiming to demonstrate the effectiveness and robustness of SAWG in practical engineering scenarios.

To assess the applicability of SAWG to practical constrained optimization, this study further considers eight classical engineering design problems, including the tension/compression spring [40,43], pressure vessel [36,40], welded beam [36,38], gear train [44], rolling element bearing [45], cantilever beam [36,39], planetary gear train [46], and robot gripper problems [46]. These tasks cover continuous, discrete, and mixed decision variables, while simultaneously involving nonlinear, inequality, equality, and strongly coupled constraints. This paper provides the problem definition for each engineering problem, including objective function, constraint, and variable range, and presents the experimental results of SAWG and other comparative algorithms. Finally, it provides an analysis of the experimental conclusions.

### 5.1. Tension/Compression Spring Design Problem

The Tension/Compression Spring Design (TCSD) problem is a continuous constrained optimization problem. The objective of this problem is to minimize the volume (V) of a coil spring under a constant tension/compression load. The problem involves three design variables:
Number of active coils of the spring:$P=x_{1},$$x_{1}\in \left[2,15\right]$Diameter of the winding:$D=x_{2},$$x_{2}\in \left[0.25,1.3\right]$Diameter of the wire:$d=x_{3},$$x_{3}\in \left[0.05,2\right]$

The mathematical formulation of the problem is as follows.

(39) $$\begin{array}{c} \begin{array}{cc} \mathrm{Minimize} & f\left(\vec{x}\right)=\left(x_{3}+2\right)x_{2}x_{1}^{2}\\ \mathrm{subject}\;\mathrm{to} & g_{1}\left(\vec{x}\right)=1-\frac{x_{2}^{3}x_{3}}{71875x_{1}^{4}}\leq 0,\\ & g_{2}\left(\vec{x}\right)=1-\frac{140.45x_{1}}{x_{2}^{2}x_{3}}\leq 0,\\ & g_{3}\left(\vec{x}\right)=\frac{x_{1}+x_{2}}{1.5}-1\leq 0,\\ & g_{4}\left(\vec{x}\right)=\frac{4x_{2}^{2}-x_{1}x_{2}}{12566\left(x_{2}x_{1}^{3}-x_{1}^{4}\right)}+\frac{1}{5108x_{1}^{2}}-1\leq 0,\\ & 2\leq x_{1}\leq 15,\;0.25\leq x_{2}\leq 1.3,\;0.05\leq x_{3}\leq 2. \end{array} \end{array}$$

For the tension spring design problem, Table 3 present the best solutions and statistical performance of SAWG in comparison with nine other metaheuristic algorithms. Overall, SAWG exhibits a pronounced competitive advantage in terms of both solution quality and robustness. As reported in Table 3, SAWG achieves the best mean objective value of 1.273 × $10^{-2}$, which is identical to the minimum optimal values obtained by the strongest competitors, such as SOO and PGA. This result indicates that SAWG can consistently identify near-optimal feasible solutions under stringent nonlinear constraints.

### 5.2. Pressure Vessel Design Problem

The pressure vessel design problem aims to minimize the total manufacturing cost while satisfying structural safety requirements. The primary design variables include the inner radius R, the head thickness $T_{h}$, the length of the cylindrical section L, and the shell thickness $T_{s}$. The problem is constrained by multiple structural requirements, including stress limitations prescribed by the ASME code, internal pressure constraints, and minimum thickness specifications. The mathematical model of the pressure vessel design problem is given as follows.

Minimize

(40) $$\begin{array}{cc} f(\vec{x}) & =0.6224\;x_{1}x_{3}x_{4}+1.7781\;x_{2}x_{3}^{2}+3.1661\;x_{1}^{2}x_{4}+19.84\;x_{1}^{2}x_{3}, \end{array}$$

subject to

(41) $$\begin{array}{cc} g_{1}\left(\vec{x}\right) & =-x_{1}+0.0193x_{3}\leq 0,\\ g_{2}\left(\vec{x}\right) & =-x_{2}+0.00954x_{3}\leq 0,\\ g_{3}\left(\vec{x}\right) & =-\pi x_{3}^{2}x_{4}-\frac{4}{3}\pi x_{3}^{3}+1,296,000\leq 0,\\ g_{4}\left(\vec{x}\right) & =x_{4}-240\leq 0,\\ 0\leq x_{1} & \leq 99,\;0\leq x_{2}\leq 99,\;10\leq x_{3}\leq 200,\;10\leq x_{4}\leq 200. \end{array}$$

For the pressure vessel design problem, SAWG consistently demonstrates excellent robustness across independent runs while delivering highly competitive solution quality. As reported in Table 4, SAWG achieves the global optimal objective value of 7.535 × $10^{2}$, which is comparable to those obtained by several strong competitors, while maintaining an exceptionally small standard deviation.

### 5.3. Welded Beam Design Problem

The welded beam design problem aims to determine the optimal geometric dimensions of a welded beam subjected to external loading. The objective is to minimize the manufacturing cost while satisfying multiple structural constraints, including stress and deflection requirements. This problem is formulated as an engineering optimization task with four design variables, where $\left[x_{1},x_{2},x_{3},x_{4}\right]$ represent the weld thickness, weld length, beam height, and beam width, respectively. The mathematical model of the welded beam design problem is presented as follows.

Minimize

(42) $$\begin{array}{cc} f(\vec{x}) & =1.10471\;x_{1}^{2}x_{2}+0.04811\;x_{3}x_{4}\left(14.0+x_{2}\right), \end{array}$$

subject to

(43) $$\begin{array}{cc} g_{1}\left(\vec{x}\right) & =\tau \left(\vec{x}\right)-\tau_{\max}\leq 0,\\ g_{2}\left(\vec{x}\right) & =\sigma \left(\vec{x}\right)-\sigma_{\max}\leq 0,\\ g_{3}\left(\vec{x}\right) & =\delta \left(\vec{x}\right)-\delta_{\max}\leq 0,\\ g_{4}\left(\vec{x}\right) & =x_{1}-x_{4}\leq 0,\\ g_{5}\left(\vec{x}\right) & =P-P_{c}\left(\vec{x}\right)\leq 0,\\ g_{6}\left(\vec{x}\right) & =0.125-x_{1}\leq 0,\\ g_{7}\left(\vec{x}\right) & =1.10471x_{1}^{2}+0.04811x_{3}x_{4}\left(14.0+x_{2}\right)-5.0\leq 0,\\ 0.1 & \leq x_{1}\leq 2,\;0.1\leq x_{2}\leq 10,\\ 0.1 & \leq x_{3}\leq 10,\;0.1\leq x_{4}\leq 2 \end{array}$$

where

(44) $$\begin{array}{cc} M_{R} & =P\left(L+x_{2}^{2}\right),\\ R & =\sqrt{\frac{x_{2}^{2}}{4}+{\left(\frac{x_{1}+x_{3}}{2}\right)}^{2}},\\ \tau^{\prime } & =\frac{P}{\sqrt{x_{1}x_{2}}},\;\tau^{\prime \prime }=\frac{M_{R}J}{M},\\ \sigma (\vec{x}) & =\frac{6PL}{x_{4}x_{3}^{2}},\;\delta \left(\vec{x}\right)=\frac{6PL^{3}}{Ex_{3}^{2}x_{4}},\\ \tau (\vec{x}) & =\sqrt{{\left(\tau^{\prime }\right)}^{2}+2\tau^{\prime }\tau^{\prime \prime }\left(\frac{x_{2}}{R}\right)+{\left(\tau^{\prime \prime }\right)}^{2}},\\ J & =2\left[\sqrt{x_{1}x_{2}}\left(x_{2}^{2}+{\left(x_{1}+\frac{x_{3}}{2}\right)}^{2}\right)\right],\\ P_{c}\left(\vec{x}\right) & =\frac{4.013E}{L^{2}}\sqrt{\frac{x_{3}^{2}x_{4}^{6}}{36}}\left(1-\frac{x_{3}}{2L}\sqrt{\frac{E}{4G}}\right). \end{array}$$

As shown in Table 5, SAWG achieves the best overall performance in terms of the mean objective value (3.482 × $10^{-10}$) while also obtaining the global optimal solution (2.701 × $10^{-12}$). Compared with the competing algorithms, SAWG consistently delivers lower average manufacturing costs, demonstrating its superior search capability within the narrow feasible regions that are typical of structural optimization problems.

### 5.4. Gear Train Design Problem

The gear train design problem aims to minimize the cost associated with the gear transmission ratio and represents a typical unconstrained discrete optimization problem in mechanical engineering. The optimization objective is defined in terms of the gear ratio, which is expressed as the ratio between the angular velocity of the output shaft and that of the input shaft. Gear train design plays a critical role in mechanical transmission systems, where optimizing the transmission ratio can not only enhance transmission efficiency but also reduce manufacturing costs and operational energy consumption. In this formulation, the numbers of teeth on the gears, denoted by $x_{1},x_{2},x_{3},$ and $x_{4}$, are treated as discrete design variables. The mathematical model of the gear train design problem is presented as follows.

Minimize

(45) $$\begin{array}{cc} f(\vec{x}) & ={\left(\frac{1}{6.931}-\frac{x_{3}x_{2}}{x_{1}x_{4}}\right)}^{2}, \end{array}$$

subject to

(46) $$\begin{array}{cc} 12 & \leq x_{i}\leq 60,\;i=1,2,3,4. \end{array}$$

As shown in Table 6, SAWG consistently maintained highly competitive average performance across multiple independent runs and was able to continuously obtain the globally optimal solution with a mean of 3.482 × $10^{-10}$.

### 5.5. Rolling Element Bearing Design

The rolling element bearing design problem aims to maximize the fatigue life of the bearing, which is a critical factor governing its reliability and operational performance. In this formulation, ten design variables are used to define the key geometric parameters of the bearing, while nine constraints are imposed to regulate dynamic load capacity, material characteristics, and operating conditions. The optimization process explicitly accounts for contact stress, lubrication conditions, and fatigue life models to ensure that the bearing meets the minimum required service life. The objective function is defined to maximize the L10 rated life, subject to manufacturing and operational constraints. The mathematical model of the rolling element bearing design problem is presented as follows.

**Table 3:** Tension spring design problem.

	GWO	TOC	PSO	HHO	PGA	RIME	SAO	SOO	WOA	SAWG
Mean	1.281 × $10^{-2}$	1.394 × $10^{-2}$	1.321 × $10^{-2}$	1.352 × $10^{-2}$	1.292 × $10^{-2}$	1.618 × $10^{-2}$	1.342 × $10^{-2}$	1.273 × $10^{-2}$	1.351 × $10^{-2}$	1.273 × $10^{-2}$
Std	1.304 × $10^{-4}$	4.493 × $10^{-3}$	9.340 × $10^{-4}$	8.643 × $10^{-4}$	5.866 × $10^{-4}$	2.033 × $10^{-3}$	1.201 × $10^{-3}$	7.613 × $10^{-5}$	1.029 × $10^{-3}$	7.793 × $10^{-5}$
Best	1.269 × $10^{-2}$	1.267 × $10^{-2}$	1.267 × $10^{-2}$	1.270 × $10^{-2}$	1.267 × $10^{-2}$	1.274 × $10^{-2}$	1.268 × $10^{-2}$	1.267 × $10^{-2}$	1.267 × $10^{-2}$	1.267 × $10^{-2}$
Worst	1.322 × $10^{-2}$	3.045 × $10^{-2}$	1.777 × $10^{-2}$	1.642 × $10^{-2}$	1.587 × $10^{-2}$	1.802 × $10^{-2}$	1.774 × $10^{-2}$	1.296 × $10^{-2}$	1.639 × $10^{-2}$	1.312 × $10^{-2}$
X1	1.064 × $10^{1}$	1.130 × $10^{1}$	7.359	6.114	9.486	3.629	8.151	1.068 × $10^{1}$	6.808	1.306 × $10^{1}$
X2	3.830 × $10^{-1}$	4.132 × $10^{-1}$	4.787 × $10^{-1}$	5.300 × $10^{-1}$	4.140 × $10^{-1}$	7.767 × $10^{-1}$	4.829 × $10^{-1}$	3.735 × $10^{-1}$	5.141 × $10^{-1}$	3.352 × $10^{-1}$
X3	5.264 × $10^{-2}$	5.309 × $10^{-2}$	5.608 × $10^{-2}$	5.783 × $10^{-2}$	5.376 × $10^{-2}$	6.475 × $10^{-2}$	5.598 × $10^{-2}$	5.233 × $10^{-2}$	5.719 × $10^{-2}$	5.072 × $10^{-2}$

**Table 4:** Pressure vessel design problem.

	GWO	TOC	PSO	HHO	PGA	RIME	SAO	SOO	WOA	SAWG
Mean	8.035 × $10^{2}$	8.028 × $10^{2}$	7.535 × $10^{2}$	1.272 × $10^{3}$	7.535 × $10^{2}$	7.558 × $10^{2}$	9.259 × $10^{2}$	7.535 × $10^{2}$	8.362 × $10^{2}$	7.535 × $10^{2}$
Std	1.873 × $10^{2}$	1.875 × $10^{2}$	1.156 × $10^{-13}$	2.995 × $10^{2}$	1.156 × $10^{-13}$	7.750	3.179 × $10^{2}$	1.156 × $10^{-13}$	2.253 × $10^{2}$	4.483 × $10^{-5}$
Best	7.535 × $10^{2}$	7.535 × $10^{2}$	7.535 × $10^{2}$	7.537 × $10^{2}$	7.535 × $10^{2}$	7.535 × $10^{2}$	7.535 × $10^{2}$	7.535 × $10^{2}$	7.535 × $10^{2}$	7.535 × $10^{2}$
Worst	1.493 × $10^{3}$	1.493 × $10^{3}$	7.535 × $10^{2}$	1.559 × $10^{3}$	7.535 × $10^{2}$	7.959 × $10^{2}$	1.493 × $10^{3}$	7.535 × $10^{2}$	1.503 × $10^{3}$	7.535 × $10^{2}$
X1	3.028 × $10^{-2}$	3.027 × $10^{-2}$	0.000	1.847 × $10^{-1}$	3.738 × $10^{-19}$	0.000	1.059 × $10^{-1}$	0.000	4.440 × $10^{-2}$	5.269 × $10^{-11}$
X2	7.313 × $10^{-5}$	2.011 × $10^{-10}$	0.000	8.019 × $10^{-12}$	2.024 × $10^{-20}$	0.000	1.614 × $10^{-18}$	0.000	0.000	0.000
X3	4.198 × $10^{1}$	4.198 × $10^{1}$	4.032 × $10^{1}$	5.518 × $10^{1}$	4.032 × $10^{1}$	4.038 × $10^{1}$	4.613 × $10^{1}$	4.032 × $10^{1}$	4.302 × $10^{1}$	4.032 × $10^{1}$
X4	1.873 × $10^{2}$	1.873 × $10^{2}$	2.000 × $10^{2}$	7.620 × $10^{1}$	2.000 × $10^{2}$	1.993 × $10^{2}$	1.557 × $10^{2}$	2.000 × $10^{2}$	1.781 × $10^{2}$	2.000 × $10^{2}$

**Table 5:** Welded beam design problem.

	GWO	TOC	PSO	HHO	PGA	RIME	SAO	SOO	WOA	SAWG
Mean	1.695	1.811	1.695	2.006	1.693	2.409	1.719	1.705	8.124	1.692
Std	2.058 × $10^{-3}$	2.191 × $10^{-1}$	1.468 × $10^{-2}$	2.470 × $10^{-1}$	4.293 × $10^{-3}$	4.711 × $10^{-1}$	1.128 × $10^{-1}$	4.409 × $10^{-2}$	2.344 × $10^{1}$	1.259 × $10^{-4}$
Best	1.693	1.692	1.692	1.701	1.692	1.731	1.692	1.692	1.728	1.692
Worst	1.704	2.496	1.773	2.729	1.715	3.487	2.282	1.872	1.256 × $10^{2}$	1.693
X1	2.058 × $10^{-1}$	1.909 × $10^{-1}$	2.063 × $10^{-1}$	2.103 × $10^{-1}$	2.067 × $10^{-1}$	3.749 × $10^{-1}$	2.140 × $10^{-1}$	2.050 × $10^{-1}$	4.274 × $10^{-1}$	2.064 × $10^{-1}$
X2	3.239	4.095	3.210	4.582	3.217	2.422	3.168	3.233	2.910	3.222
X3	9.040	9.361	9.069	8.528	9.031	6.657	8.923	9.108	7.101	9.037
X4	2.058 × $10^{-1}$	2.044 × $10^{-1}$	2.056 × $10^{-1}$	2.461 × $10^{-1}$	2.060 × $10^{-1}$	4.157 × $10^{-1}$	2.133 × $10^{-1}$	2.058 × $10^{-1}$	4.624 × $10^{-1}$	2.057 × $10^{-1}$

Maximize:

(47) $$\begin{array}{cc} f(\vec{x}) & =\left\{\begin{array}{cc} f_{c}\;Z^{2/3}\;D_{b}^{1.8}, & \mathrm{if}\;D_{b}\leq 25.4\;\mathrm{mm},\\ 3.647\;f_{c}\;Z^{2/3}\;D_{b}^{1.4}, & \mathrm{if}\;D_{b}>25.4\;\mathrm{mm}, \end{array}\right. \end{array}$$

subject to

(48) $$\begin{array}{cc} g_{1}\left(\vec{x}\right) & =\frac{\phi_{o}}{2\sin^{-1}\left(D_{b}/D_{m}\right)}\geq 0,\\ g_{2}\left(\vec{x}\right) & =2D_{b}-K_{D_{\min}}\left(D-d\right)\geq 0,\\ g_{3}\left(\vec{x}\right) & =K_{D_{\max}}\left(D-d\right)-2D_{b}\geq 0,\\ g_{4}\left(\vec{x}\right) & =D_{m}-\left(0.5-e\right)\left(D+d\right)\geq 0,\\ g_{5}\left(\vec{x}\right) & =\left(0.5+e\right)\left(D+d\right)-D_{m}\geq 0,\\ g_{6}\left(\vec{x}\right) & =D_{m}-0.5\left(D+d\right)\geq 0,\\ g_{7}\left(\vec{x}\right) & =0.5\left(D-D_{m}-D_{b}\right)-\varepsilon D_{b}\geq 0,\\ g_{8}\left(\vec{x}\right) & =\zeta B_{w}-D_{b}\leq 0,\\ g_{9}\left(\vec{x}\right) & =f_{i}\geq 0.515,\\ g_{10}\left(\vec{x}\right) & =f_{o}\geq 0.515,\\ 0.5\;(D+d) & \leq D_{m}\leq 0.6\;\left(D+d\right),\\ 0.15\;(D-d) & \leq D_{b}\leq 0.45\;\left(D-d\right),\\ 4\leq Z\leq 50,\;0.515 & \leq f_{i}\leq 0.6,\;0.515\leq f_{o}\leq 0.6,\\ 0.4\leq K_{D_{\min}} & \leq 0.5,\;0.6\leq K_{D_{\max}}\leq 0.7,\\ 0.3\leq \varepsilon \leq 0.4,\;0.02 & \leq e\leq 0.1,\;0.6\leq \zeta \leq 0.85. \end{array}$$

where

(49) $$\begin{array}{cc} \vec{x} & =\left[x_{1},x_{2},x_{3},x_{4},x_{5},x_{6},x_{7},x_{8},x_{9},x_{10}\right]=\left[D_{m},D_{b},Z,f_{i},f_{o},K_{D\min},K_{D\max},\varepsilon ,e,\zeta \right].\\ f_{c} & =37.91{\left\{1+{\left[1.04{\left(\frac{1-\gamma }{1+\gamma }\right)}^{1.72}{\left(\frac{f_{i}\left(2f_{o}-1\right)}{f_{o}\left(2f_{i}-1\right)}\right)}^{0.41}\right]}^{10/3}\right\}}^{-0.3}\left[\frac{\gamma^{0.3}{\left(1-\gamma \right)}^{1.39}}{f_{o}{\left(1+\gamma \right)}^{1/3}}\right]{\left[\frac{2f_{i}}{2f_{i}-1}\right]}^{0.41}.\\ \varphi_{o} & =2\pi -2\arccos\left(\frac{{\left(\frac{D-d}{2}-3\left(\frac{T}{4}\right)\right)}^{2}+{\left(\frac{D}{2}-\frac{T}{4}-D_{b}\right)}^{2}-{\left(\frac{d}{2}+\frac{T}{4}\right)}^{2}}{2\left(\frac{D-d}{2}-3\left(\frac{T}{4}\right)\right)\left(\frac{D}{2}-\frac{T}{4}-D_{b}\right)}\right),\\ T & =D-d-2D_{b},\;B_{w},\;D=160,\;d=90,\;r_{i}=r_{o}=11.033. \end{array}$$

Table 7 shows that, in the rolling element bearing design problem, the mean and best values obtained by SAWG are identical and the variance is zero, indicating strong stability on high-dimensional problems with strict constraints.

### 5.6. Cantilever Beam Design Problem

The cantilever beam design problem seeks a minimum-weight design by optimizing the geometric dimensions of the beam components under a set of prescribed constraints. The beam is composed of five hollow segments with square cross-sections, and each segment is characterized by a structural parameter with constant thickness, leading to five design variables in total. The task is to simultaneously determine the optimal values of these five parameters so that the resulting cantilever beam satisfies all constraints while achieving the minimum possible weight. The mathematical formulation of the cantilever beam design problem is presented as follows.

Minimize

(50) $$\begin{array}{cc} f(\vec{x}) & =0.0624\left(x_{1}+x_{2}+x_{3}+x_{4}+x_{5}\right), \end{array}$$

subject to

(51) $$\begin{array}{cc} g(\vec{x}) & =\frac{61}{x_{1}^{3}}+\frac{37}{x_{2}^{3}}+\frac{19}{x_{3}^{3}}+\frac{7}{x_{4}^{3}}+\frac{1}{x_{5}^{3}}-1\leq 0,\\ 0.01 & \leq x_{i}\leq 100,\;i=1,2,\ldots ,5. \end{array}$$

Table 8 shows that, in the cantilever beam design problem, SAWG achieves one of the best objective values with extremely small variance, indicating that it can still maintain effective control near flat optimal regions.

### 5.7. Planetary Gear Train Design

The planetary gear train design problem aims to minimize the maximum error of the gear ratio in automotive transmission systems. Reducing the maximum gear ratio error improves transmission accuracy and reliability while mitigating wear and energy losses. The mathematical formulation of the planetary gear train design problem is presented as follows:

(52) $$\begin{array}{cc} f(\vec{x}) & =\max\left|i_{k}-i_{0k}\right|,\;k=\left\{1,2,\ldots ,R\right\}, \end{array}$$

subject to

(53) $$\begin{array}{cc} g_{1}\left(\vec{x}\right) & =m_{3}\left(N_{6}+2.5\right)-D_{\max}\leq 0,\\ g_{2}\left(\vec{x}\right) & =m_{1}\left(N_{1}+N_{2}\right)+m_{1}\left(N_{2}+2\right)-D_{\max}\leq 0,\\ g_{3}\left(\vec{x}\right) & =m_{3}\left(N_{4}+N_{5}\right)+m_{3}\left(N_{5}+2\right)-D_{\max}\leq 0,\\ g_{4}\left(\vec{x}\right) & =\left|m_{1}\left(N_{1}+N_{2}\right)-m_{3}\left(N_{6}-N_{3}\right)\right|-m_{1}-m_{3}\leq 0,\\ g_{5}\left(\vec{x}\right) & =-\left(N_{1}+N_{2}\right)\sin\left(\pi /p\right)+N_{2}+2+\delta_{22}\leq 0,\\ g_{6}\left(\vec{x}\right) & =-\left(N_{6}-N_{3}\right)\sin\left(\pi /p\right)+N_{3}+2+\delta_{33}\leq 0,\\ g_{7}\left(\vec{x}\right) & =-\left(N_{4}+N_{5}\right)\sin\left(\pi /p\right)+N_{5}+2+\delta_{55}\leq 0,\\ g_{8}\left(\vec{x}\right) & ={\left(N_{3}+N_{5}+2+\delta_{35}\right)}^{2}-{\left(N_{6}-N_{3}\right)}^{2}-{\left(N_{4}+N_{5}\right)}^{2}\\ \; & \;+2\left(N_{6}-N_{3}\right)\left(N_{4}+N_{5}\right)\cos\left(\frac{2\pi }{p}-\beta \right)\leq 0,\\ g_{9}\left(\vec{x}\right) & =N_{4}-N_{6}+2N_{5}+2\delta_{56}+4\leq 0,\\ g_{10}\left(\vec{x}\right) & =2N_{3}-N_{6}+N_{4}+2\delta_{34}+4\leq 0,\\ h_{1}\left(\vec{x}\right) & =\frac{N_{6}-N_{4}}{p}\in Z,\\ p & \in \left\{3,4,5\right\},\;N_{i}\in Z,\\ m_{1} & \in \{1.75,2.0,2.25,2.5,2.75,3.0\},\\ m_{3} & \in \{1.75,2.0,2.25,2.5,2.75,3.0\},\\ 17 & \leq N_{1}\leq 96,\;14\leq N_{2}\leq 54,\;14\leq N_{3}\leq 51,\\ 17 & \leq N_{4}\leq 46,\;14\leq N_{5}\leq 51,\;48\leq N_{6}\leq 124 \end{array}$$

where

(54) $$\begin{array}{cc} \vec{x} & =\left[x_{1},x_{2},x_{3},x_{4},x_{5},x_{6},x_{7},x_{8},x_{9}\right]=\left(p,N_{6},N_{5},N_{4},N_{3},N_{2},N_{1},m_{2},m_{1}\right),\\ i_{1} & =\frac{N_{6}}{N_{4}},\;i_{01}=3.11,\\ i_{2} & =\frac{N_{6}\left(N_{1}N_{3}+N_{2}N_{4}\right)}{N_{1}N_{3}\left(N_{6}-N_{4}\right)},\;i_{02}=1.84,\\ i_{R} & =-3.11,\;I_{R}=-\frac{N_{2}N_{6}}{N_{1}N_{3}}i_{02},\\ \delta_{22} & =\delta_{33}=\delta_{55}=\delta_{35}=\delta_{56}=0.5,\;D_{\max}=220,\\ \beta & =\cos^{-1}\left(\frac{{\left(N_{4}+N_{5}\right)}^{2}+{\left(N_{6}-N_{3}\right)}^{2}-{\left(N_{3}+N_{5}\right)}^{2}}{2\left(N_{6}-N_{3}\right)\left(N_{4}+N_{5}\right)}\right). \end{array}$$

Table 9 indicates that, in the planetary gear train design problem, SAWG achieves the best results in both mean objective value and worst-case performance, while converging stably to a high-quality solution.

### 5.8. Robot Gripper Problem

The robot gripper design problem features a relatively complex optimization objective, which seeks to minimize the difference between the minimum and maximum gripping forces generated by the gripper. Robot grippers are essential components in industrial automation and precision manufacturing, and their design directly influences the accuracy and efficiency of robotic operations. The mathematical formulation of the robot gripper design problem is presented as follows.

Minimize

(55) $$\begin{array}{c} f\left(\vec{x}\right)=-\min F_{k}\left(\vec{x},z\right)+\max F_{k}\left(\vec{x},z\right), \end{array}$$

subject to

(56) $$\begin{array}{cc} g_{1}\left(\vec{x}\right) & =-Y_{\min}+y\left(\vec{x},Z_{\max}\right)\leq 0,\\ g_{2}\left(\vec{x}\right) & =-y(\vec{x},Z_{\max})\leq 0,\\ g_{3}\left(\vec{x}\right) & =Y_{\max}-y\left(\vec{x},0\right)\leq 0,\\ g_{4}\left(\vec{x}\right) & =y\left(\vec{x},0\right)-Y_{G}\leq 0,\\ g_{5}\left(\vec{x}\right) & =l^{2}+e^{2}-{\left(a+b\right)}^{2}\leq 0,\\ g_{6}\left(\vec{x}\right) & =b^{2}-{\left(a-e\right)}^{2}-{\left(l-Z_{\max}\right)}^{2}\leq 0,\\ g_{7}\left(\vec{x}\right) & =Z_{\max}-l\leq 0,\\ 0 & \leq e\leq 50,\;100\leq c\leq 200,\\ 10 & \leq f,a,b\leq 150,\\ 1 & \leq \delta \leq 3.14,\;100\leq l\leq 300 \end{array}$$

where

(57) $$\begin{array}{cc} \vec{x} & =\left[x_{1},x_{2},x_{3},x_{4},x_{5},x_{6},x_{7}\right]=\left[a,b,c,e,f,l,\delta \right],\\ F_{k} & =\frac{Pb\sin(\alpha +\beta )}{2c\cos(\alpha )},\\ \beta & =\cos^{-1}\left(\frac{b^{2}+g^{2}-a^{2}}{2bg}\right)-\phi ,\\ \alpha & =\cos^{-1}\left(\frac{a^{2}+g^{2}-b^{2}}{2ag}\right)+\phi ,\\ \phi & =\tan^{-1}\left(\frac{e}{l-z}\right),\\ g & =\sqrt{e^{2}+{\left(z-l\right)}^{2}},\\ y(\vec{x},z) & =2\left(f+e+c\sin(\beta +\delta )\right),\\ Y_{\min} & =50,\;Y_{\max}=100,\;Y_{G}=150,\;Z_{\max}=100,\;P=100. \end{array}$$

Table 10 further shows that, in the robot gripper problem, SAWG obtains the lowest mean objective value and a near-zero best value with extremely small variance.

Taken together, these results suggest that the advantage of SAWG does not rely on any single problem structure but is instead reflected in the coordinated enhancement of feasibility maintenance, search stability, and solution quality across different variable types, constraint forms, and objective landscapes.

**Figure 9:**
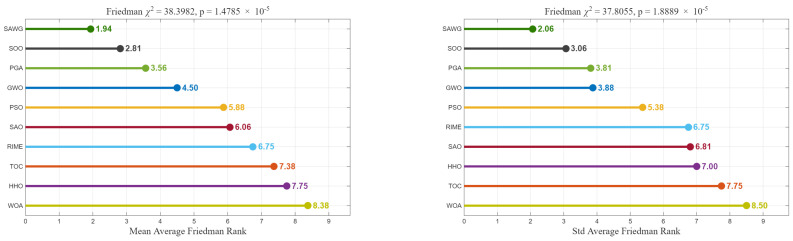
Lollipop chart of engineering test cases.

**Figure 10:**
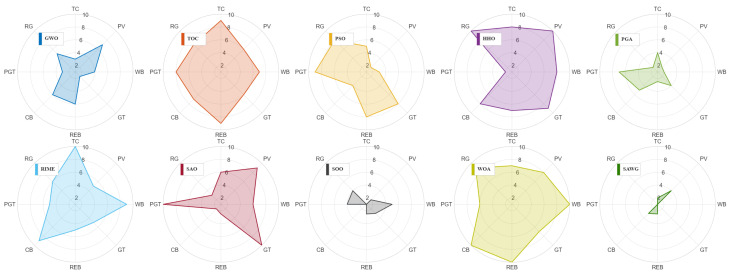
Radar charts of engineering test cases. TC: tension/compression spring; PV: pressure vessel; WB: welded beam; GT: gear train; REB: rolling element bearing; CB: cantilever beam; PGT: planetary gear train; RG: robot gripper.

Finally, on the basis of the above eight typical constrained engineering optimization problems, Friedman ranking analyses were conducted on the mean and standard deviation results of SAWG and the competing algorithms, as illustrated in Figure 9 and Figure 10. SAWG consistently maintained the highest Friedman score and had the smallest radar chart area. This show that SAWG simultaneously achieves superior solution quality, higher stability, and stronger cross-problem generalization ability across a variety of heterogeneous constrained engineering optimization tasks. This indicates that SAWG possesses favorable effectiveness and application potential in practical engineering optimization scenarios.

**Table 6:** Gear train design problem.

	GWO	TOC	PSO	HHO	PGA	RIME	SAO	SOO	WOA	SAWG
Mean	5.185 × $10^{-10}$	3.255 × $10^{-9}$	3.894 × $10^{-9}$	4.435 × $10^{-9}$	1.254 × $10^{-9}$	2.054 × $10^{-9}$	7.074 × $10^{-9}$	7.810 × $10^{-10}$	3.568 × $10^{-9}$	3.482 × $10^{-10}$
Std	5.073 × $10^{-10}$	4.243 × $10^{-9}$	6.543 × $10^{-9}$	7.895 × $10^{-9}$	8.742 × $10^{-10}$	4.880 × $10^{-9}$	8.768 × $10^{-9}$	7.994 × $10^{-10}$	6.890 × $10^{-9}$	4.543 × $10^{-10}$
Best	2.701 × $10^{-12}$	2.701 × $10^{-12}$	2.701 × $10^{-12}$	2.308 × $10^{-11}$	2.308 × $10^{-11}$	2.308 × $10^{-11}$	2.308 × $10^{-11}$	2.701 × $10^{-12}$	2.701 × $10^{-12}$	2.701 × $10^{-12}$
Worst	1.362 × $10^{-9}$	1.827 × $10^{-8}$	2.726 × $10^{-8}$	2.726 × $10^{-8}$	3.300 × $10^{-9}$	2.726 × $10^{-8}$	2.726 × $10^{-8}$	2.358 × $10^{-9}$	2.726 × $10^{-8}$	1.362 × $10^{-9}$
X1	4.600 × $10^{1}$	5.200 × $10^{1}$	4.500 × $10^{1}$	4.500 × $10^{1}$	5.100 × $10^{1}$	4.900 × $10^{1}$	4.800 × $10^{1}$	4.800 × $10^{1}$	4.800 × $10^{1}$	4.800 × $10^{1}$
X2	1.800 × $10^{1}$	2.200 × $10^{1}$	1.700 × $10^{1}$	1.700 × $10^{1}$	2.000 × $10^{1}$	1.900 × $10^{1}$	2.100 × $10^{1}$	1.700 × $10^{1}$	1.800 × $10^{1}$	1.900 × $10^{1}$
X3	1.900 × $10^{1}$	1.800 × $10^{1}$	2.000 × $10^{1}$	2.000 × $10^{1}$	2.300 × $10^{1}$	1.900 × $10^{1}$	1.600 × $10^{1}$	2.000 × $10^{1}$	2.000 × $10^{1}$	1.800 × $10^{1}$
X4	4.700 × $10^{1}$	4.800 × $10^{1}$	4.700 × $10^{1}$	4.800 × $10^{1}$	5.300 × $10^{1}$	4.700 × $10^{1}$	4.600 × $10^{1}$	4.500 × $10^{1}$	4.800 × $10^{1}$	4.700 × $10^{1}$

**Table 7:** Rolling element bearing design problem.

	GWO	TOC	PSO	HHO	PGA	RIME	SAO	SOO	WOA	SAWG
Mean	−3.047 × $10^{5}$	−3.047 × $10^{5}$	−3.047 × $10^{5}$	−3.047 × $10^{5}$	−3.047 × $10^{5}$	−3.047 × $10^{5}$	−3.047 × $10^{5}$	−3.047 × $10^{5}$	−3.040 × $10^{5}$	−3.047 × $10^{5}$
Std	6.000	3.600 × $10^{1}$	2.800 × $10^{1}$	1.700 × $10^{1}$	0.000	2.000	0.000	0.000	3.967E+03	0.000
Best	−3.047 × $10^{5}$	−3.047 × $10^{5}$	−3.047 × $10^{5}$	−3.047 × $10^{5}$	−3.047 × $10^{5}$	−3.047 × $10^{5}$	−3.047 × $10^{5}$	−3.047 × $10^{5}$	−3.047 × $10^{5}$	−3.047 × $10^{5}$
Worst	−3.047 × $10^{5}$	−3.046 × $10^{5}$	−3.046 × $10^{5}$	−3.046 × $10^{5}$	−3.047 × $10^{5}$	−3.047 × $10^{5}$	−3.047 × $10^{5}$	−3.047 × $10^{5}$	−2.830 × $10^{5}$	−3.047 × $10^{5}$
X1	1.250 × $10^{2}$	1.250 × $10^{2}$	1.250 × $10^{2}$	1.250 × $10^{2}$	1.250 × $10^{2}$	1.250 × $10^{2}$	1.250 × $10^{2}$	1.250 × $10^{2}$	1.250 × $10^{2}$	1.250 × $10^{2}$
X2	2.586 × $10^{1}$	2.586 × $10^{1}$	2.586 × $10^{1}$	2.586 × $10^{1}$	2.586 × $10^{1}$	2.585 × $10^{1}$	2.586 × $10^{1}$	2.586 × $10^{1}$	2.584 × $10^{1}$	2.586 × $10^{1}$
X3	5.000 × $10^{1}$	5.000 × $10^{1}$	5.000 × $10^{1}$	5.000 × $10^{1}$	5.000 × $10^{1}$	5.000 × $10^{1}$	5.000 × $10^{1}$	5.000 × $10^{1}$	5.000 × $10^{1}$	5.000 × $10^{1}$
X4	5.150 × $10^{-1}$	5.150 × $10^{-1}$	5.150 × $10^{-1}$	5.150 × $10^{-1}$	5.150 × $10^{-1}$	5.150 × $10^{-1}$	5.150 × $10^{-1}$	5.150 × $10^{-1}$	5.150 × $10^{-1}$	5.150 × $10^{-1}$
X5	5.185 × $10^{-1}$	5.460 × $10^{-1}$	5.292 × $10^{-1}$	5.198 × $10^{-1}$	5.150 × $10^{-1}$	5.150 × $10^{-1}$	5.150 × $10^{-1}$	5.150 × $10^{-1}$	5.428 × $10^{-1}$	5.150 × $10^{-1}$
X6	4.479 × $10^{-1}$	4.600 × $10^{-1}$	4.499 × $10^{-1}$	4.524 × $10^{-1}$	4.468 × $10^{-1}$	4.329 × $10^{-1}$	4.563 × $10^{-1}$	4.459 × $10^{-1}$	4.332 × $10^{-1}$	4.384 × $10^{-1}$
X7	7.000 × $10^{-1}$	7.000 × $10^{-1}$	7.000 × $10^{-1}$	7.000 × $10^{-1}$	7.000 × $10^{-1}$	7.000 × $10^{-1}$	7.000 × $10^{-1}$	7.000 × $10^{-1}$	7.000 × $10^{-1}$	7.000 × $10^{-1}$
X8	3.000 × $10^{-1}$	3.000 × $10^{-1}$	3.000 × $10^{-1}$	3.000 × $10^{-1}$	3.000 × $10^{-1}$	3.000 × $10^{-1}$	3.000 × $10^{-1}$	3.000 × $10^{-1}$	3.033 × $10^{-1}$	3.000 × $10^{-1}$
X9	4.938 × $10^{-2}$	7.207 × $10^{-2}$	6.847 × $10^{-2}$	6.422 × $10^{-2}$	5.525 × $10^{-2}$	5.693 × $10^{-2}$	6.452 × $10^{-2}$	5.788 × $10^{-2}$	6.023 × $10^{-2}$	4.100 × $10^{-2}$
X10	7.355 × $10^{-1}$	7.500 × $10^{-1}$	7.204 × $10^{-1}$	6.801 × $10^{-1}$	7.526 × $10^{-1}$	6.481 × $10^{-1}$	7.096 × $10^{-1}$	7.106 × $10^{-1}$	6.902 × $10^{-1}$	7.273 × $10^{-1}$

**Table 8:** Cantilever beam design problem.

	GWO	TOC	PSO	HHO	PGA	RIME	SAO	SOO	WOA	SAWG
Mean	1.340	1.343	1.340	1.344	1.340	1.376	1.340	1.340	1.637	1.340
Std	5.962 × $10^{-5}$	5.382 × $10^{-3}$	2.629 × $10^{-6}$	2.096 × $10^{-3}$	1.061 × $10^{-4}$	5.758 × $10^{-2}$	1.866 × $10^{-9}$	1.989 × $10^{-12}$	1.753 × $10^{-1}$	1.733 × $10^{-7}$
Best	1.340	1.340	1.340	1.340	1.340	1.342	1.340	1.340	1.405	1.340
Worst	1.340	1.357	1.340	1.348	1.340	1.658	1.340	1.340	2.133	1.340
X1	6.015	6.000	6.015	6.008	6.009	6.204	6.016	6.016	6.285	6.016
X2	5.309	5.324	5.309	5.272	5.314	5.438	5.309	5.309	5.784	5.309
X3	4.496	4.525	4.494	4.552	4.493	4.532	4.494	4.494	6.177	4.494
X4	3.498	3.479	3.502	3.509	3.504	3.693	3.501	3.501	4.701	3.501
X5	2.155	2.198	2.152	2.191	2.154	2.188	2.152	2.153	3.293	2.152

**Table 9:** Planetary gear train design problem.

	GWO	TOC	PSO	HHO	PGA	RIME	SAO	SOO	WOA	SAWG
Mean	2.412 × $10^{-1}$	6.346 × $10^{1}$	7.168 × $10^{1}$	2.397 × $10^{-1}$	2.641 × $10^{-1}$	2.416 × $10^{-1}$	1.800 × $10^{3}$	2.416 × $10^{-1}$	2.482 × $10^{-1}$	2.394 × $10^{-1}$
Std	4.067 × $10^{-3}$	3.454 × $10^{2}$	3.911 × $10^{2}$	2.156 × $10^{-3}$	5.932 × $10^{-2}$	6.140 × $10^{-3}$	3.662 × $10^{3}$	8.372 × $10^{-3}$	1.750 × $10^{-2}$	3.758 × $10^{-3}$
Best	2.353 × $10^{-1}$	2.355 × $10^{-1}$	2.368 × $10^{-1}$	2.359 × $10^{-1}$	2.378 × $10^{-1}$	2.355 × $10^{-1}$	2.394 × $10^{-1}$	2.353 × $10^{-1}$	2.359 × $10^{-1}$	2.350 × $10^{-1}$
Worst	2.500 × $10^{-1}$	1.892 × $10^{3}$	2.142 × $10^{3}$	2.445 × $10^{-1}$	5.645 × $10^{-1}$	2.689 × $10^{-1}$	9.000 × $10^{3}$	2.782 × $10^{-1}$	3.346 × $10^{-1}$	2.485 × $10^{-1}$
X1	3.588 × $10^{1}$	2.827 × $10^{1}$	4.436 × $10^{1}$	2.529 × $10^{1}$	3.360 × $10^{1}$	3.795 × $10^{1}$	1.840 × $10^{1}$	3.826 × $10^{1}$	2.354 × $10^{1}$	3.027 × $10^{1}$
X2	2.520 × $10^{1}$	1.989 × $10^{1}$	2.470 × $10^{1}$	2.295 × $10^{1}$	2.177 × $10^{1}$	2.528 × $10^{1}$	1.629 × $10^{1}$	2.640 × $10^{1}$	2.112 × $10^{1}$	2.260 × $10^{1}$
X3	1.928 × $10^{1}$	1.897 × $10^{1}$	1.617 × $10^{1}$	1.990 × $10^{1}$	1.740 × $10^{1}$	1.837 × $10^{1}$	1.430 × $10^{1}$	1.971 × $10^{1}$	1.777 × $10^{1}$	1.658 × $10^{1}$
X4	3.033 × $10^{1}$	2.656 × $10^{1}$	3.032 × $10^{1}$	2.517 × $10^{1}$	2.804 × $10^{1}$	2.995 × $10^{1}$	1.778 × $10^{1}$	3.133 × $10^{1}$	2.224 × $10^{1}$	2.520 × $10^{1}$
X5	1.781 × $10^{1}$	1.585 × $10^{1}$	1.669 × $10^{1}$	1.998 × $10^{1}$	1.623 × $10^{1}$	1.718 × $10^{1}$	1.424 × $10^{1}$	1.871 × $10^{1}$	1.733 × $10^{1}$	1.539 × $10^{1}$
X6	8.789 × $10^{1}$	7.652 × $10^{1}$	8.873 × $10^{1}$	7.298 × $10^{1}$	8.324 × $10^{1}$	8.706 × $10^{1}$	5.169 × $10^{1}$	9.102 × $10^{1}$	6.471 × $10^{1}$	7.279 × $10^{1}$
X7	2.637	3.062	2.048	2.230	3.144	2.353	1.608	2.655	1.624	2.068
X8	1.525	1.700	1.718	1.462	1.614	1.654	1.133	1.935	1.391	1.547
X9	1.414	1.268	1.760	1.247	1.461	1.438	1.064	1.466	1.311	1.227

**Table 10:** Robot gripper design problem.

	GWO	TOC	PSO	HHO	PGA	RIME	SAO	SOO	WOA	SAWG
Mean	2.977	3.106	3.370	1.309 × $10^{1}$	1.012 × $10^{-16}$	3.020	1.690	1.969	3.479	7.568 × $10^{-17}$
Std	1.249	2.434	6.033 × $10^{-1}$	2.188 × $10^{1}$	3.616 × $10^{-17}$	2.246	1.946	1.430	3.162	8.888 × $10^{-18}$
Best	7.346 × $10^{-17}$	8.074 × $10^{-17}$	2.547	1.486 × $10^{-16}$	7.274 × $10^{-17}$	7.344 × $10^{-17}$	7.274 × $10^{-17}$	1.064 × $10^{-16}$	1.456 × $10^{-16}$	7.274 × $10^{-17}$
Worst	4.095	1.129 × $10^{1}$	4.314	8.051 × $10^{1}$	1.797 × $10^{-16}$	1.036 × $10^{1}$	5.179	3.658	9.292	1.128 × $10^{-16}$
X1	1.443 × $10^{2}$	1.390 × $10^{2}$	1.491 × $10^{2}$	1.379 × $10^{2}$	9.702 × $10^{1}$	1.326 × $10^{2}$	1.225 × $10^{2}$	1.294 × $10^{2}$	1.232 × $10^{2}$	9.987 × $10^{1}$
X2	1.229 × $10^{2}$	1.149 × $10^{2}$	1.193 × $10^{2}$	1.069 × $10^{2}$	3.521 × $10^{1}$	9.375 × $10^{1}$	6.989 × $10^{1}$	9.435 × $10^{1}$	7.511 × $10^{1}$	3.806 × $10^{1}$
X3	1.868 × $10^{2}$	1.801 × $10^{2}$	1.893 × $10^{2}$	1.428 × $10^{2}$	1.920 × $10^{2}$	1.900 × $10^{2}$	1.766 × $10^{2}$	1.900 × $10^{2}$	1.392 × $10^{2}$	1.960 × $10^{2}$
X4	1.125 × $10^{1}$	6.634	2.874 × $10^{1}$	1.665 × $10^{1}$	0.000	1.685 × $10^{1}$	1.237 × $10^{1}$	1.308 × $10^{1}$	1.490 × $10^{1}$	0.000
X5	1.029 × $10^{2}$	1.048 × $10^{2}$	1.136 × $10^{2}$	8.014 × $10^{1}$	9.237 × $10^{1}$	1.279 × $10^{2}$	7.245 × $10^{1}$	8.453 × $10^{1}$	7.648 × $10^{1}$	8.408 × $10^{1}$
X6	1.278 × $10^{2}$	1.064 × $10^{2}$	1.182 × $10^{2}$	1.613 × $10^{2}$	1.000 × $10^{2}$	1.311 × $10^{2}$	1.075 × $10^{2}$	1.053 × $10^{2}$	1.302 × $10^{2}$	1.000 × $10^{2}$
X7	2.277	2.255	2.527	2.523	2.091	2.562	2.037	2.142	2.312	1.911

Taken together, the numerical benchmark experiments and engineering case studies demonstrate that SAWG is able to maintain a favorable balance between exploration and exploitation across different optimization scenarios, thereby exhibiting strong competitiveness and general applicability. Meanwhile, SAWG consistently preserves stable convergence characteristics and high-quality solutions under diverse heterogeneous problem settings, further suggesting that its performance advantage is not limited to standard benchmark tests but extends to a certain degree of cross-scenario generalization. Overall, SAWG not only achieves strong performance on theoretical benchmark problems but also shows a favorable capacity for handling complex real-world engineering design tasks.

## 6. Discussion and Future Directions

### 6.1. Conclusions

In this study, a novel adaptive gradient-guided metaheuristic optimization algorithm, termed Self-Adaptive AdamW-Guided Optimization (SAWG), is proposed to address the core challenges commonly encountered in complex black-box optimization, including the unavailability of gradient information and the inherent trade-off between search efficiency and stability. Unlike conventional swarm intelligence algorithms that primarily rely on purely random operators or empirical heuristic rules, SAWG introduces gradient-inspired guidance while preserving the generality and robustness of metaheuristic optimization, thereby systematically integrating gradient-like learning mechanisms into population-based search.

The key characteristics of SAWG can be summarized as follows. First, to cope with the absence of true gradient information in black-box optimization, a population-statistics-based pseudo-gradient estimation mechanism is constructed, providing stable and computable directional guidance for the search process. Second, an AdamW-style adaptive update strategy is incorporated into the population evolution, embedding first- and second-order moment estimation together with weight decay regularization to dynamically regulate both search direction and step size. Third, a stagnation-aware adaptive control mechanism is designed to identify potential efficiency degradation during the search process and to effectively alleviate premature convergence through targeted exploration compensation. These mechanisms operate synergistically within a unified framework, enabling SAWG to achieve an adaptive balance between exploration and exploitation.

To systematically evaluate the performance of SAWG, qualitative analyses are first conducted, demonstrating that SAWG exhibits more reasonable and stable behavioral characteristics in terms of search trajectory structure, smoothness of fitness evolution, and dynamic exploration–exploitation regulation, thereby validating the effectiveness of its gradient-guided update mechanism. Subsequently, extensive experiments are carried out on representative and widely adopted CEC benchmark suites, including CEC2017 and CEC2020, under multiple dimensional settings. The experimental results indicate that SAWG consistently achieves significant or highly competitive performance advantages over a range of strong and competitive comparison algorithms across multiple evaluation criteria, including best and mean fitness values, convergence behavior, stability, and statistical significance.

Furthermore, to assess its practical applicability in real-world scenarios, SAWG is successfully applied to several classical engineering design optimization problems. The results demonstrate that, even in the presence of nonlinear constraints, complex feasible regions, and strong variable coupling, SAWG can reliably obtain high-quality solutions while maintaining consistent advantages in both accuracy and robustness. These findings clearly indicate that SAWG not only performs competitively on theoretical benchmark problems but also exhibits strong generalization capability and application potential in practical engineering optimization.

### 6.2. Future Work

Although SAWG has demonstrated encouraging performance, there remains considerable room for further extension and enhancement. Future research will focus on the following directions:

- *Lightweight improvement strategies for SAWG:* Examples include adopting a fixed recursive depth, an adaptive recursive depth, dimension sampling, or recursive early-stopping mechanisms, with the aim of further reducing the algorithmic complexity from the current $O(TND^{2})$ to a level close to O(TND), which is typical of standard swarm intelligence optimization algorithms.
- *Algorithmic enhancement:* Further improving pseudo-gradient construction and adaptive update strategies, for example, by incorporating more locally sensitive population information or multi-scale learning mechanisms, to enhance search efficiency in high-dimensional and highly complex problems.
- *Extended application scenarios:* Extending SAWG to multi-objective, large-scale, and dynamic optimization problems, in conjunction with more advanced constraint-handling techniques, to further broaden its applicability to complex real-world problems.

In summary, SAWG provides a new paradigm for the systematic integration of gradient learning concepts and metaheuristic optimization. By introducing adaptive and learnable search mechanisms into gradient-free environments, SAWG offers a robust and efficient solution for both complex benchmark functions and practical engineering optimization problems, while also serving as a valuable reference for future research on learning-driven metaheuristic algorithms.

## Figures and Tables

**Table 1:** Wilcoxon rank-sum test (with Holm correction) results summary on the CEC2017 test functions.

CEC2017	Type	Pairwise Comparison Results (+/≈/−)								
		GWO	TOC	PSO	HHO	PGA	RIME	SAO	SOO	WOA
10Dim	Unimodal	2/0/0	2/0/0	1/0/1	2/0/0	1/0/1	2/0/0	1/0/1	0/0/2	2/0/0
	Multimodal	7/0/0	7/0/0	3/2/2	7/0/0	5/1/1	6/1/0	5/0/2	4/3/0	7/0/0
	Hybrid	10/0/0	9/1/0	9/1/0	10/0/0	9/1/0	8/2/0	9/1/0	9/0/1	10/0/0
	Composite	9/1/0	8/2/0	8/2/0	9/1/0	7/2/1	7/3/0	7/3/0	7/3/0	9/1/0
	Total	28/1/0	26/3/0	21/5/3	28/1/0	22/4/3	23/6/0	22/4/3	20/6/3	28/1/0
30Dim	Unimodal	2/0/0	2/0/0	2/0/0	2/0/0	2/0/0	2/0/0	2/0/0	2/0/0	2/0/0
	Multimodal	6/1/0	7/0/0	6/1/0	7/0/0	7/0/0	6/0/1	6/1/0	6/0/1	7/0/0
	Hybrid	10/0/0	10/0/0	10/0/0	10/0/0	9/1/0	7/3/0	7/3/0	7/3/0	10/0/0
	Composite	10/0/0	10/0/0	10/0/0	10/0/0	8/2/0	9/1/0	7/2/1	10/0/0	10/0/0
	Total	28/1/0	29/0/0	28/1/0	29/0/0	26/3/0	24/4/1	22/6/1	25/3/1	29/0/0
50Dim	Unimodal	2/0/0	2/0/0	2/0/0	2/0/0	2/0/0	2/0/0	1/1/0	2/0/0	2/0/0
	Multimodal	6/1/0	7/0/0	6/1/0	7/0/0	7/0/0	6/1/0	6/1/0	6/0/1	7/0/0
	Hybrid	10/0/0	10/0/0	10/0/0	10/0/0	8/2/0	9/1/0	8/2/0	10/0/0	10/0/0
	Composite	9/1/0	10/0/0	9/1/0	10/0/0	8/2/0	8/2/0	7/3/0	9/0/1	10/0/0
	Total	27/2/0	29/0/0	27/2/0	29/0/0	25/4/0	25/4/0	22/7/0	27/0/2	29/0/0
100Dim	Unimodal	2/0/0	2/0/0	2/0/0	2/0/0	2/0/0	2/0/0	2/0/0	2/0/0	2/0/0
	Multimodal	7/0/0	7/0/0	6/1/0	7/0/0	7/0/0	6/1/0	7/0/0	6/1/0	7/0/0
	Hybrid	10/0/0	10/0/0	10/0/0	10/0/0	8/2/0	10/0/0	7/3/0	10/0/0	10/0/0
	Composite	10/0/0	10/0/0	10/0/0	10/0/0	10/0/0	10/0/0	9/1/0	9/1/0	10/0/0
	Total	29/0/0	29/0/0	28/1/0	29/0/0	27/2/0	28/1/0	25/4/0	27/2/0	29/0/0

**Table 2:** Wilcoxon rank-sum test (with Holm correction) results summary on the CEC2020 test functions.

CEC2020	Type	Pairwise Comparison Results (+/≈/−)								
		GWO	TOC	PSO	HHO	PGA	RIME	SAO	SOO	WOA
10Dim	Unimodal	1/0/0	1/0/0	1/0/0	1/0/0	1/0/0	1/0/0	1/0/0	0/0/1	1/0/0
	Multimodal	2/0/1	3/0/0	3/0/0	2/0/1	3/0/0	2/1/0	3/0/0	0/2/1	2/0/1
	Hybrid	3/0/0	3/0/0	3/0/0	3/0/0	3/0/0	3/0/0	3/0/0	3/0/0	3/0/0
	Composite	2/1/0	2/1/0	2/1/0	2/1/0	2/1/0	2/1/0	2/1/0	2/1/0	3/0/0
	Total	8/1/1	9/1/0	9/1/0	8/1/1	9/1/0	8/2/0	9/1/0	5/3/2	9/0/1
30Dim	Unimodal	1/0/0	1/0/0	1/0/0	1/0/0	1/0/0	1/0/0	1/0/0	1/0/0	1/0/0
	Multimodal	2/0/1	3/0/0	3/0/0	2/0/1	3/0/0	2/1/0	3/0/0	1/0/2	2/0/1
	Hybrid	3/0/0	3/0/0	3/0/0	3/0/0	3/0/0	3/0/0	3/0/0	3/0/0	3/0/0
	Composite	3/0/0	3/0/0	3/0/0	3/0/0	2/1/0	3/0/0	1/1/1	3/0/0	3/0/0
	Total	9/0/1	10/0/0	10/0/0	9/0/1	9/1/0	9/1/0	8/1/1	8/0/2	9/0/1
50Dim	Unimodal	1/0/0	1/0/0	1/0/0	1/0/0	1/0/0	1/0/0	0/1/0	1/0/0	1/0/0
	Multimodal	2/0/1	2/0/1	2/1/0	2/0/1	3/0/0	2/1/0	2/1/0	1/0/2	2/0/1
	Hybrid	3/0/0	3/0/0	3/0/0	3/0/0	3/0/0	3/0/0	3/0/0	3/0/0	3/0/0
	Composite	2/1/0	3/0/0	2/1/0	3/0/0	2/1/0	1/2/0	1/2/0	2/0/1	3/0/0
	Total	8/1/1	9/0/1	8/2/0	9/0/1	9/1/0	7/3/0	6/4/0	7/0/3	9/0/1
100Dim	Unimodal	1/0/0	1/0/0	1/0/0	1/0/0	1/0/0	1/0/0	1/0/0	1/0/0	1/0/0
	Multimodal	2/0/1	2/0/1	3/0/0	2/0/1	3/0/0	2/1/0	3/0/0	1/1/1	2/0/1
	Hybrid	3/0/0	3/0/0	3/0/0	3/0/0	3/0/0	3/0/0	3/0/0	3/0/0	3/0/0
	Composite	3/0/0	3/0/0	3/0/0	3/0/0	3/0/0	3/0/0	3/0/0	2/1/0	3/0/0
	Total	9/0/1	9/0/1	10/0/0	9/0/1	10/0/0	9/1/0	10/0/0	7/2/1	9/0/1

## Data Availability

The data used in this study must be authentic and reliable to ensure the validity and reproducibility of the experimental results. The datasets and source code used in this study are openly available at https://github.com/xieeix-code/The-Intelligent-Optimization-Algorithm-SAWG (accessed on 27 January 2026). All benchmark data sets used in this study (CEC2017 and CEC2020) are publicly available from the official CEC competition website.

## Appendix A

**Table A1:** Running results of 10 algorithms on CEC2017 (100D).

Function		GWO	TOC	PSO	HHO	PGA	RIME	SAO	SOO	WOA	SAWG
$F_{1}$	Mean	5.02 × $10^{10}$	1.02 × $10^{10}$	4.26 × $10^{10}$	3.27 × $10^{8}$	1.66 × $10^{4}$	6.78 × $10^{5}$	4.79 × $10^{7}$	7.44 × $10^{10}$	1.17 × $10^{9}$	**6.97 × $10^{3}$**
	Std	1.10 × $10^{10}$	4.76 × $10^{9}$	1.57 × $10^{10}$	3.63 × $10^{7}$	1.83 × $10^{4}$	4.01 × $10^{5}$	2.62 × $10^{8}$	1.63 × $10^{10}$	3.46 × $10^{8}$	1.01 × $10^{4}$
$F_{3}$	Mean	2.22 × $10^{5}$	2.65 × $10^{5}$	2.63 × $10^{5}$	1.37 × $10^{5}$	2.08 × $10^{5}$	9.35 × $10^{4}$	7.31 × $10^{5}$	1.89 × $10^{5}$	7.59 × $10^{5}$	**3.00 × $10^{2}$**
	Std	1.84 × $10^{4}$	1.33 × $10^{5}$	8.65 × $10^{4}$	1.80 × $10^{4}$	2.14 × $10^{4}$	1.77 × $10^{4}$	1.41 × $10^{5}$	3.78 × $10^{4}$	1.38 × $10^{5}$	8.86 × $10^{-8}$
$F_{4}$	Mean	4.85 × $10^{3}$	2.47 × $10^{3}$	5.79 × $10^{3}$	9.08 × $10^{2}$	6.47 × $10^{2}$	7.27 × $10^{2}$	6.46 × $10^{2}$	9.53 × $10^{3}$	1.52 × $10^{3}$	**4.85 × $10^{2}$**
	Std	1.60 × $10^{3}$	5.85 × $10^{2}$	3.61 × $10^{3}$	6.81 × $10^{1}$	3.46 × $10^{1}$	5.86 × $10^{1}$	2.95 × $10^{1}$	2.69 × $10^{3}$	2.00 × $10^{2}$	9.97 × $10^{1}$
$F_{5}$	Mean	1.15 × $10^{3}$	1.73 × $10^{3}$	1.02 × $10^{3}$	1.45 × $10^{3}$	1.12 × $10^{3}$	9.75 × $10^{2}$	9.24 × $10^{2}$	1.18 × $10^{3}$	1.51 × $10^{3}$	**7.16 × $10^{2}$**
	Std	6.70 × $10^{1}$	1.10 × $10^{2}$	8.31 × $10^{1}$	5.27 × $10^{1}$	9.83 × $10^{1}$	8.07 × $10^{1}$	1.23 × $10^{2}$	6.27 × $10^{1}$	8.76 × $10^{1}$	4.58 × $10^{1}$
$F_{6}$	Mean	6.37 × $10^{2}$	6.87 × $10^{2}$	6.26 × $10^{2}$	6.80 × $10^{2}$	6.42 × $10^{2}$	6.18 × $10^{2}$	6.09 × $10^{2}$	6.43 × $10^{2}$	6.87 × $10^{2}$	**6.05 × $10^{2}$**
	Std	4.99	6.37	5.16	3.25	9.56	3.90	3.46	4.03	9.85	4.30
$F_{7}$	Mean	1.98 × $10^{3}$	3.50 × $10^{3}$	1.76 × $10^{3}$	3.58 × $10^{3}$	1.80 × $10^{3}$	1.27 × $10^{3}$	1.96 × $10^{3}$	2.87 × $10^{3}$	3.33 × $10^{3}$	**1.10 × $10^{3}$**
	Std	1.38 × $10^{2}$	2.34 × $10^{2}$	3.02 × $10^{2}$	1.30 × $10^{2}$	1.50 × $10^{2}$	7.15 × $10^{1}$	9.24 × $10^{1}$	2.29 × $10^{2}$	1.62 × $10^{2}$	1.03 × $10^{2}$
$F_{8}$	Mean	1.46 × $10^{3}$	2.15 × $10^{3}$	1.35 × $10^{3}$	1.91 × $10^{3}$	1.45 × $10^{3}$	1.30 × $10^{3}$	1.21 × $10^{3}$	1.49 × $10^{3}$	1.99 × $10^{3}$	**1.00 × $10^{3}$**
	Std	5.20 × $10^{1}$	1.37 × $10^{2}$	5.90 × $10^{1}$	4.69 × $10^{1}$	1.08 × $10^{2}$	8.91 × $10^{1}$	5.72 × $10^{1}$	7.12 × $10^{1}$	9.01 × $10^{1}$	3.84 × $10^{1}$
$F_{9}$	Mean	2.80 × $10^{4}$	5.14 × $10^{4}$	2.88 × $10^{4}$	4.07 × $10^{4}$	1.43 × $10^{4}$	1.89 × $10^{4}$	1.88 × $10^{4}$	4.09 × $10^{4}$	4.52 × $10^{4}$	**2.04 × $10^{3}$**
	Std	1.17 × $10^{4}$	7.50 × $10^{3}$	1.95 × $10^{4}$	4.99 × $10^{3}$	6.24 × $10^{3}$	7.98 × $10^{3}$	7.43 × $10^{3}$	6.81 × $10^{3}$	1.17 × $10^{4}$	6.09 × $10^{2}$
$F_{10}$	Mean	1.57 × $10^{4}$	2.51 × $10^{4}$	1.46 × $10^{4}$	1.90 × $10^{4}$	1.55 × $10^{4}$	1.46 × $10^{4}$	1.54 × $10^{4}$	1.91 × $10^{4}$	2.27 × $10^{4}$	**1.40 × $10^{4}$**
	Std	3.33 × $10^{3}$	2.13 × $10^{3}$	1.52 × $10^{3}$	1.35 × $10^{3}$	1.10 × $10^{3}$	1.47 × $10^{3}$	3.32 × $10^{3}$	8.22 × $10^{3}$	2.35 × $10^{3}$	1.07 × $10^{3}$
$F_{11}$	Mean	5.57 × $10^{4}$	3.03 × $10^{4}$	1.58 × $10^{4}$	3.43 × $10^{3}$	2.24 × $10^{3}$	3.14 × $10^{3}$	1.17 × $10^{5}$	9.69 × $10^{3}$	4.95 × $10^{4}$	**1.30 × $10^{3}$**
	Std	1.41 × $10^{4}$	3.60 × $10^{4}$	1.70 × $10^{4}$	2.89 × $10^{2}$	3.24 × $10^{2}$	3.18 × $10^{2}$	4.37 × $10^{4}$	3.46 × $10^{3}$	3.07 × $10^{4}$	7.74 × $10^{1}$
$F_{12}$	Mean	9.76 × $10^{9}$	2.39 × $10^{9}$	1.52 × $10^{10}$	3.76 × $10^{8}$	4.30 × $10^{6}$	2.08 × $10^{8}$	1.24 × $10^{7}$	1.05 × $10^{10}$	1.16 × $10^{9}$	**1.24 × $10^{5}$**
	Std	3.69 × $10^{9}$	1.03 × $10^{9}$	1.06 × $10^{10}$	1.16 × $10^{8}$	1.80 × $10^{6}$	8.52 × $10^{7}$	7.27 × $10^{6}$	3.40 × $10^{9}$	4.77 × $10^{8}$	5.43 × $10^{4}$
$F_{13}$	Mean	1.16 × $10^{9}$	5.45 × $10^{7}$	1.17 × $10^{9}$	5.89 × $10^{6}$	1.51 × $10^{4}$	1.32 × $10^{5}$	**1.02 × $10^{4}$**	2.78 × $10^{8}$	1.08 × $10^{6}$	1.15 × $10^{4}$
	Std	1.19 × $10^{9}$	3.07 × $10^{7}$	1.00 × $10^{9}$	1.12 × $10^{6}$	9.63 × $10^{3}$	9.12 × $10^{4}$	6.63 × $10^{3}$	3.07 × $10^{8}$	8.45 × $10^{5}$	1.16 × $10^{4}$
$F_{14}$	Mean	6.08 × $10^{6}$	8.05 × $10^{6}$	2.16 × $10^{6}$	1.27 × $10^{6}$	4.49 × $10^{5}$	1.27 × $10^{6}$	1.76 × $10^{5}$	7.14 × $10^{5}$	3.53 × $10^{6}$	**1.10 × $10^{4}$**
	Std	3.53 × $10^{6}$	1.32 × $10^{7}$	1.32 × $10^{6}$	4.09 × $10^{5}$	2.78 × $10^{5}$	5.60 × $10^{5}$	1.16 × $10^{5}$	6.17 × $10^{5}$	1.10 × $10^{6}$	5.18 × $10^{3}$
$F_{15}$	Mean	1.13 × $10^{8}$	1.49 × $10^{7}$	3.33 × $10^{8}$	1.64 × $10^{6}$	7.91 × $10^{3}$	3.74 × $10^{4}$	**4.51 × $10^{3}$**	3.35 × $10^{6}$	3.24 × $10^{5}$	7.37 × $10^{3}$
	Std	1.30 × $10^{8}$	3.52 × $10^{7}$	5.72 × $10^{8}$	5.36 × $10^{5}$	5.97 × $10^{3}$	1.77 × $10^{4}$	4.44 × $10^{3}$	8.45 × $10^{6}$	6.22 × $10^{5}$	8.01 × $10^{3}$
$F_{16}$	Mean	5.91 × $10^{3}$	9.85 × $10^{3}$	6.07 × $10^{3}$	7.33 × $10^{3}$	5.61 × $10^{3}$	6.09 × $10^{3}$	5.19 × $10^{3}$	5.76 × $10^{3}$	1.18 × $10^{4}$	**3.52 × $10^{3}$**
	Std	5.45 × $10^{2}$	1.52 × $10^{3}$	8.78 × $10^{2}$	7.58 × $10^{2}$	6.42 × $10^{2}$	6.90 × $10^{2}$	6.80 × $10^{2}$	6.81 × $10^{2}$	1.81 × $10^{3}$	6.46 × $10^{2}$
$F_{17}$	Mean	4.75 × $10^{3}$	7.08 × $10^{3}$	6.54 × $10^{3}$	6.35 × $10^{3}$	4.99 × $10^{3}$	5.20 × $10^{3}$	4.68 × $10^{3}$	5.12 × $10^{3}$	7.64 × $10^{3}$	**3.57 × $10^{3}$**
	Std	6.70 × $10^{2}$	1.35 × $10^{3}$	1.26 × $10^{3}$	5.04 × $10^{2}$	6.09 × $10^{2}$	6.21 × $10^{2}$	5.56 × $10^{2}$	8.53 × $10^{2}$	1.23 × $10^{3}$	6.13 × $10^{2}$
$F_{18}$	Mean	4.73 × $10^{6}$	8.40 × $10^{6}$	3.11 × $10^{6}$	2.88 × $10^{6}$	1.24 × $10^{6}$	2.32 × $10^{6}$	1.56 × $10^{6}$	1.38 × $10^{6}$	3.25 × $10^{6}$	**3.25 × $10^{4}$**
	Std	2.86 × $10^{6}$	8.41 × $10^{6}$	1.43 × $10^{6}$	9.95 × $10^{5}$	7.10 × $10^{5}$	1.24 × $10^{6}$	1.83 × $10^{6}$	7.14 × $10^{5}$	1.35 × $10^{6}$	1.92 × $10^{4}$
$F_{19}$	Mean	2.97 × $10^{8}$	1.54 × $10^{7}$	3.72 × $10^{8}$	5.52 × $10^{6}$	9.77 × $10^{3}$	3.21 × $10^{4}$	**5.88 × $10^{3}$**	7.03 × $10^{6}$	2.24 × $10^{7}$	9.38 × $10^{3}$
	Std	3.82 × $10^{8}$	1.53 × $10^{7}$	4.72 × $10^{8}$	2.37 × $10^{6}$	9.25 × $10^{3}$	1.66 × $10^{4}$	3.32 × $10^{3}$	8.61 × $10^{6}$	1.93 × $10^{7}$	8.10 × $10^{3}$
$F_{20}$	Mean	4.59 × $10^{3}$	5.89 × $10^{3}$	4.85 × $10^{3}$	5.72 × $10^{3}$	4.92 × $10^{3}$	4.96 × $10^{3}$	4.80 × $10^{3}$	5.00 × $10^{3}$	6.21 × $10^{3}$	**3.95 × $10^{3}$**
	Std	7.91 × $10^{2}$	6.11 × $10^{2}$	6.56 × $10^{2}$	6.36 × $10^{2}$	6.11 × $10^{2}$	4.45 × $10^{2}$	8.87 × $10^{2}$	1.32 × $10^{3}$	5.39 × $10^{2}$	7.40 × $10^{2}$
$F_{21}$	Mean	2.95 × $10^{3}$	3.91 × $10^{3}$	3.11 × $10^{3}$	3.90 × $10^{3}$	2.98 × $10^{3}$	2.80 × $10^{3}$	2.78 × $10^{3}$	3.07 × $10^{3}$	3.96 × $10^{3}$	**2.54 × $10^{3}$**
	Std	5.70 × $10^{1}$	1.85 × $10^{2}$	1.45 × $10^{2}$	2.12 × $10^{2}$	1.08 × $10^{2}$	8.65 × $10^{1}$	7.32 × $10^{1}$	9.90 × $10^{1}$	2.49 × $10^{2}$	4.46 × $10^{1}$
$F_{22}$	Mean	1.87 × $10^{4}$	2.73 × $10^{4}$	1.73 × $10^{4}$	2.30 × $10^{4}$	1.76 × $10^{4}$	1.77 × $10^{4}$	1.69 × $10^{4}$	1.84 × $10^{4}$	2.58 × $10^{4}$	**1.58 × $10^{4}$**
	Std	3.20 × $10^{3}$	2.35 × $10^{3}$	1.36 × $10^{3}$	1.02 × $10^{3}$	1.73 × $10^{3}$	1.54 × $10^{3}$	1.84 × $10^{3}$	6.17 × $10^{3}$	2.87 × $10^{3}$	1.66 × $10^{3}$
$F_{23}$	Mean	3.56 × $10^{3}$	5.08 × $10^{3}$	4.62 × $10^{3}$	4.76 × $10^{3}$	3.36 × $10^{3}$	3.30 × $10^{3}$	3.24 × $10^{3}$	3.88 × $10^{3}$	4.87 × $10^{3}$	**3.05 × $10^{3}$**
	Std	8.35 × $10^{1}$	3.55 × $10^{2}$	2.53 × $10^{2}$	2.03 × $10^{2}$	8.28 × $10^{1}$	7.44 × $10^{1}$	6.89 × $10^{1}$	1.27 × $10^{2}$	2.62 × $10^{2}$	4.97 × $10^{1}$
$F_{24}$	Mean	4.20 × $10^{3}$	6.64 × $10^{3}$	5.90 × $10^{3}$	5.76 × $10^{3}$	3.91 × $10^{3}$	3.78 × $10^{3}$	3.73 × $10^{3}$	4.95 × $10^{3}$	6.03 × $10^{3}$	**3.58 × $10^{3}$**
	Std	1.49 × $10^{2}$	5.63 × $10^{2}$	4.41 × $10^{2}$	2.85 × $10^{2}$	1.10 × $10^{2}$	7.79 × $10^{1}$	1.04 × $10^{2}$	2.90 × $10^{2}$	3.37 × $10^{2}$	8.00 × $10^{1}$
$F_{25}$	Mean	6.55 × $10^{3}$	4.98 × $10^{3}$	5.01 × $10^{3}$	3.55 × $10^{3}$	3.32 × $10^{3}$	3.39 × $10^{3}$	3.32 × $10^{3}$	9.50 × $10^{3}$	3.99 × $10^{3}$	**3.26 × $10^{3}$**
	Std	9.57 × $10^{2}$	5.42 × $10^{2}$	1.27 × $10^{3}$	8.14 × $10^{1}$	6.71 × $10^{1}$	6.72 × $10^{1}$	7.72 × $10^{1}$	1.04 × $10^{3}$	1.45 × $10^{2}$	6.04 × $10^{1}$
$F_{26}$	Mean	1.47 × $10^{4}$	3.21 × $10^{4}$	2.07 × $10^{4}$	2.28 × $10^{4}$	1.24 × $10^{4}$	1.13 × $10^{4}$	1.02 × $10^{4}$	2.15 × $10^{4}$	3.28 × $10^{4}$	**9.06 × $10^{3}$**
	Std	1.11 × $10^{3}$	5.02 × $10^{3}$	4.53 × $10^{3}$	5.94 × $10^{3}$	1.31 × $10^{3}$	8.92 × $10^{2}$	8.56 × $10^{2}$	2.43 × $10^{3}$	3.42 × $10^{3}$	8.52 × $10^{2}$
$F_{27}$	Mean	4.04 × $10^{3}$	4.70 × $10^{3}$	4.24 × $10^{3}$	4.14 × $10^{3}$	3.61 × $10^{3}$	3.61 × $10^{3}$	**3.45 × $10^{3}$**	4.59 × $10^{3}$	5.05 × $10^{3}$	3.47 × $10^{3}$
	Std	1.72 × $10^{2}$	5.11 × $10^{2}$	3.60 × $10^{2}$	2.88 × $10^{2}$	8.05 × $10^{1}$	7.17 × $10^{1}$	4.71 × $10^{1}$	2.03 × $10^{2}$	6.57 × $10^{2}$	6.72 × $10^{1}$
$F_{28}$	Mean	8.16 × $10^{3}$	5.66 × $10^{3}$	1.02 × $10^{4}$	3.59 × $10^{3}$	3.39 × $10^{3}$	3.49 × $10^{3}$	3.42 × $10^{3}$	1.30 × $10^{4}$	4.41 × $10^{3}$	**3.29 × $10^{3}$**
	Std	1.06 × $10^{3}$	9.82 × $10^{2}$	3.50 × $10^{3}$	3.74 × $10^{1}$	3.32 × $10^{1}$	3.69 × $10^{1}$	2.94 × $10^{1}$	1.79 × $10^{3}$	1.99 × $10^{2}$	1.03 × $10^{2}$
$F_{29}$	Mean	8.17 × $10^{3}$	1.28 × $10^{4}$	7.57 × $10^{3}$	9.18 × $10^{3}$	6.56 × $10^{3}$	7.18 × $10^{3}$	6.27 × $10^{3}$	8.92 × $10^{3}$	1.47 × $10^{4}$	**5.06 × $10^{3}$**
	Std	7.43 × $10^{2}$	1.42 × $10^{3}$	1.37 × $10^{3}$	8.47 × $10^{2}$	5.47 × $10^{2}$	5.37 × $10^{2}$	4.27 × $10^{2}$	9.53 × $10^{2}$	1.62 × $10^{3}$	5.43 × $10^{2}$
$F_{30}$	Mean	1.13 × $10^{9}$	1.78 × $10^{8}$	1.45 × $10^{9}$	3.08 × $10^{7}$	1.33 × $10^{4}$	4.38 × $10^{6}$	2.17 × $10^{4}$	2.56 × $10^{8}$	3.85 × $10^{8}$	**9.81 × $10^{3}$**
	Std	1.36 × $10^{9}$	1.83 × $10^{8}$	1.39 × $10^{9}$	8.43 × $10^{6}$	5.43 × $10^{3}$	1.79 × $10^{6}$	1.80 × $10^{4}$	1.76 × $10^{8}$	1.63 × $10^{8}$	3.67 × $10^{3}$
	Rank First	0	0	0	0	0	0	4	0	0	**25**

*F_i_* denotes the *i*th benchmark function in the CEC2017 benchmark suite. Mean and Std indicate the mean and standard deviation over 30 independent runs, respectively. The best result for each function is shown in bold.

**Table A2:** Parameter settings and experimental protocol.

Item	Setting
Population size	30
Independent runs	30
Benchmark dimensions	10, 30, 50, and 100
Maximum function evaluations	10,000 for quantitative benchmark experiments
Qualitative analysis budget	2000 function evaluations
Statistical test	Wilcoxon rank-sum test with Holm correction; Friedman test
Implementation platform	MATLAB 2025b
Source code (Accessed on 27 January 2026)	https://github.com/xieeix-code/The-Intelligent-Optimization-Algorithm-SAWG.
